# Implementation of Time-Averaged Restraints with UNRES Coarse-Grained Model of Polypeptide Chains

**DOI:** 10.1021/acs.jctc.4c01504

**Published:** 2025-01-24

**Authors:** Nguyen Truong Co, Cezary Czaplewski, Emilia A. Lubecka, Adam Liwo

**Affiliations:** † Faculty of Chemistry, University of Gdańsk, Fahrenheit Union of Universities, ul. Wita Stwosza 63, 80-308 Gdańsk, Poland; ‡ Faculty of Electronics, Telecommunications and Informatics, Gdańsk University of Technology, Fahrenheit Union of Universities in Gdańsk, ul. G. Narutowicza 11/12, 80-233 Gdańsk, Poland

## Abstract

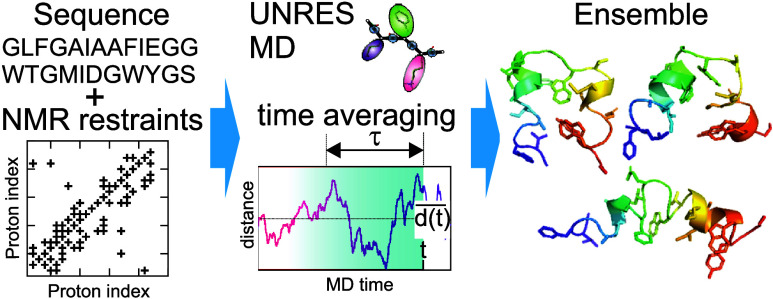

Time-averaged restraints from nuclear magnetic resonance (NMR) measurements have been implemented in the UNRES coarse-grained model of polypeptide chains in order to develop a tool for data-assisted modeling of the conformational ensembles of multistate proteins, intrinsically disordered proteins (IDPs) and proteins with intrinsically disordered regions (IDRs), many of which are essential in cell biology. A numerically stable variant of molecular dynamics with time-averaged restraints has been introduced, in which the total energy is conserved in sections of a trajectory in microcanonical runs, the bath temperature is maintained in canonical runs, and the time-average-restraint-force components are scaled up with the length of the memory window so that the restraints affect the simulated structures. The new approach restores the conformational ensembles used to generate ensemble-averaged distances, as demonstrated with synthetic restraints. The approach results in a better fitting of the ensemble-averaged interproton distances to those determined experimentally for multistate proteins and proteins with intrinsically disordered regions, which puts it at an advantage over all-atom approaches with regard to the determination of the conformational ensembles of proteins with diffuse structures, owing to a faster and more robust conformational search.

## Introduction

Proteins in solution are dynamic structures. Typically, loops are the regions with high mobility because they often contain substrate- or ligand-binding sites, the mobility being thus required for functioning.^1,2^ Multistate proteins such as, e.g., molecular chaperones,^3,4^ as well as intrinsically disordered proteins (IDPs) and proteins with intrinsically disordered regions (IDRs)^5−7^ constitute an important part of every organism’s proteosome. Therefore, instead of a fixed structure of a protein in solution, its dynamic ensemble should be considered.

Nuclear Magnetic Resonance (NMR) is a powerful technique for the determination of protein solution structures.^8^ Protein-structure determination by NMR results in a bundle of conformations, consistent with the dynamic nature of proteins in solution. The conformations can be closely related, divergent in part, if the structure contains flexible regions, or even several alternative structural ensembles are obtained for multistate proteins. NMR structure determination is based on the conformational search with a given force field subject to experimental restraints, of which the distance restraints (usually between protons) or those imposed on dihedral angles are the most common.

The nature of NMR measurements implies that the restraints correspond to time- and ensemble-averaged quantities.^9,10^ The first feature arises from a millisecond-scale mixing time of measuring the Nuclear Overhauser Effect that is the main source of distance restraints, while the second one from averaging over the whole solution ensemble. The determination of the structures of bioactive peptides by using time-averaged distance and angular restraints has a long history^11−19^ but was not applied to proteins except for small ones.^20^ Ensemble averaging can be performed in two manners: by including the restraints at simulation time through replica averaging^21−24^ or by postsimulation reweighting of the resulting conformational ensemble.^25−29^ Ensemble reweighting has found more practical applications and has been implemented, e.g., in the Xplor-NIH package.^28^

The extent and speed of conformational search, which is crucial for modeling the conformational ensembles of flexible proteins, can be increased tremendously with coarse-grained models, in which atoms are merged into extended interaction sites.^30−33^ Apart from reducing the number of interaction sites, their advantage is the dilatation of the apparent time scale with respect to the all-atom or laboratory time scale due to removing explicit solvent molecules (for implicit-solvent models) and internal friction. This dilatation can amount to 3 orders of magnitude for heavily coarse-grained models.^34,35^ The resolution of coarse-grained models is lower compared to all-atom ones but still seems reasonable, especially for flexible proteins.

In this study we developed an improved version of the time-averaged-restraint algorithm proposed by Torda et al.^11^ and Bonvin et al.^14^ and implemented it in the molecular dynamics (MD) with the UNRES coarse-grained model of polypeptide chains.^36,37^ Owing to its physics-based derivation, in particular to our recently developed theory of coarse-graining,^38^ UNRES is able to model protein structures and dynamics with considerable accuracy.^37,39^ We used our recently developed ESCASA algorithm^40^ to estimate proton positions from coarse-grained geometry analytically. We found that the method developed in this work restores the conformational ensembles from which synthetic restraints were generated and, for multistate proteins and proteins with disordered regions, gives ensembles better satisfying the experimental restraints than the respective ensembles deposited in the Protein Data Bank (PDB).^41^

## Methods

### UNRES Model of Polypeptide Chains and its Implementation

**Figure 1:**
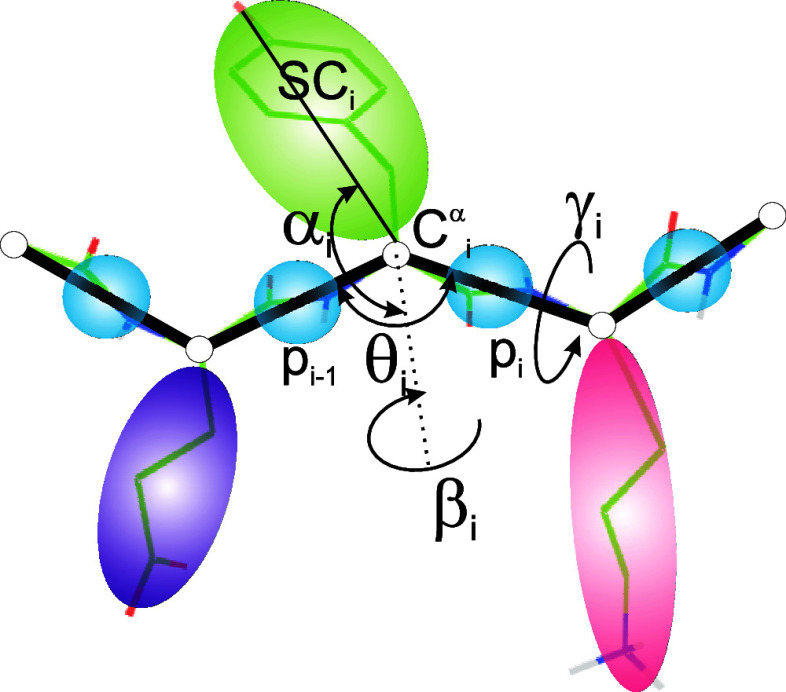
UNRES model of polypeptide chains. The interaction sites are united peptide groups located between the consecutive α-carbon atoms (light-blue spheres) and united side chains attached to the α-carbon atoms (spheroids with different colors and dimensions). The backbone geometry of the simplified polypeptide chain is defined by the C^α^···C^α^···C^α^ virtual-bond angles θ (θ_*i*_ has the vertex at C_*i*_^α^) and the C^α^···C^α^···C^α^···C^α^ virtual-bond-dihedral angles γ (γ_*i*_ has the axis passing trough C_*i*_^α^ and C_*i*+1_^α^). The local geometry of the *i*th side-chain center is defined by the zenith angle α_*i*_ (the angle between the bisector of the respective angle θ_*i*_ and the C_*i*_^α^···SC_*i*_ vector) and the azimuth angle β_*i*_ (the angle of counterclockwise rotation of the C_*i*_^α^···SC_*i*_ vector about the bisector from the C_*i*–1_^α^···C_*i*_^α^···C_*i*+1_^α^ plane, starting from C_*i*–1_^α^). For illustration, the bonds of the all-atom chains, except for those to the hydrogen atoms connected with the carbon atoms, are superposed on the coarse-grained picture. Reproduced with permission from Zaborowski et al., J. Chem. Inf. Model., 55, 2050 (2015). Copyright 2015 American Chemical Society.

In the UNRES model,^36,37^ a polypeptide chain is represented as the trace of α-carbon (C^α^) atoms, which are not interaction sites, linked with backbone virtual bonds, with the peptide groups (p) located halfway between the consecutive C^α^ atoms and the side chains (SC) attached to the C^α^ atoms with virtual bonds, these two kinds of objects being the interaction sites, as shown in Figure 1. The peptide-group sites represent the C′, O, N, and H backbone atoms and the side-chain sites represent the side-chain and the C^α^ and H^α^ atoms (Figure 1). The Cartesian coordinates of the C^α^ atoms and those of the SC centers are used as variables.^35^ The energy function consists of site–site, local, and correlation potentials, the latter accounting for the coupling between the backbone-local and backbone-electrostatic interactions.^36−38,42^ Most of the site–site interaction potentials have the axial and not the spherical symmetry. This feature and the presence of correlation terms are responsible for good performance of UNRES despite aggressive coarse graining. The UNRES effective energy function depends on temperature, which reflects the fact that it originates from the potential of mean force of polypeptide chains in water.^43^ Details of the energy function and its derivation are described elsewhere.^36−38,42^ In this work, we used the NEWCT-9P variant of the UNRES force field calibrated, by means of the maximum likelihood method, with a set of 9 proteins with different structural classes.^44^

The conformational search with UNRES is carried out with the use of MD, which has been implemented in our earlier work.^34,45^ This implementation has further been extended^46^ to multiplexed replica exchange molecular dynamics (MREMD),^47^ with which the exploration of protein conformational space is more efficient. In MREMD, multiple trajectories are run at each temperature, which results in a more thorough search of the conformational space. To determine the weights of the conformations of an ensemble found by MREMD at the desired temperatures and, thereby, the ensemble averages, we use the binless variant of the weighted histogram analysis method (WHAM),^48^ which was adapted to the temperature-dependent UNRES energy function in our earlier work.^49^ The code has been parallelized in our earlier work^50^ with the Message Passing Interface (MPI) libraries and, recently, heavily optimized and parallelized in the hybrid MPI/OpenMP mode.^35^ Further, we ported the code to single^51^ and multiple^52^ Graphical Processor Units (GPUs).

### Restraints from NMR with UNRES

Restraints are included as penalty terms added to the UNRES energy function. In this study, we imposed restraints on the distances between protons of different residues, on the C^α^···C^α^···C^α^ backbone-virtual-bond angles θ and on the C^α^···C^α^···C^α^···C^α^ backbone virtual-bond dihedral angles γ. The θ and γ angles are shown in Figure 1. The proton coordinates to compute the distances are estimated analytically from the coarse-grained geometry by means of the ESCASA algorithm.^40,53^ The angular restraints are derived from those on the backbone ϕ and ψ angles, by using eqs 10 and 22 from the paper by Nishikawa et al.^54^ The extended energy function, including the penalty terms, is given by eq 1.
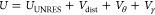
1where *U*_UNRES_ is the UNRES energy function, *V*_dist_ is the interproton-distance penalty term, and *V*_θ_ and *V*_γ_ are the respective angular penalty terms. These restraint potentials are given by eqs 2 – 4, respectively.^55−57^
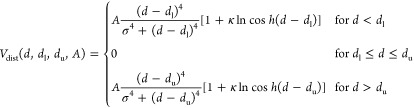
2where *d* is a proton–proton distance (estimated from the coarse-grained coordinates), *d*_l_ and *d*_u_ are the lower and upper distance boundaries, respectively, which are taken from NMR data, σ is the thickness of the restraint-well wall, *A* is the depth of the restraint-potential well, and κ is the slope of the restraint potential at large distances.^57^ As in our earlier work,^53^ we set κ = 0.01 to provide a small gradient driving at the desired distances but not to force the fulfillment of all restraints, which are likely to be contradictory for a system that consists of many interconverting conformations. We set σ = 1 Å, while *A* varied depending on the required restraint strength. It should be noted that σ determines the size of the attractor region of the penalty function. The value of σ established in our earlier work,^53^ in which the restraints were not time averaged, was σ = 0.5 Å. However, in this work which concerns time-averaged restraints, we found that the attractor region should be larger and therefore increased σ.
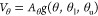
3
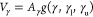
4with
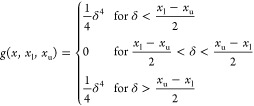
5
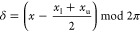
6where θ_l_, θ_u_, γ_l_, and γ_u_ are the lower and upper boundaries on the virtual-bond angles θ and virtual-bond-dihedral angles γ, respectively (which are calculated from the boundaries on the ϕ and ψ backbone dihedral angles). *A*_θ_ = 1 kcal/mol in this work and *A*_γ_ = 5 kcal/mol in this work are the restraint-potential-well depths.

The distances and angles in the penalty functions can be calculated from a single conformation or be time- or replica-averaged. In this work, we consider time-averaged restraints, which are described in section “Time-Averaged Restraints”.

In our earlier work^53^ we extended the distance-penalty term to treat ambiguous NOE restraints. This feature was not used in the current work because all assignments were unambiguous.

### Time-Averaged Restraints

In the time-average-restraint method, the distances and angles from the simulated structures present in eqs 2–4 are averaged over a memory window^11−19^ with an exponential memory function, as expressed by eq 7.
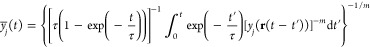
7where *y*_*j*_(**r**(*t*)) is the *j*th restrained quantity, which depends on the coordinates that describe the geometry of the system (C^α^ atoms and side-chain centers in this work) collected in vector **r**(*t*) at time *t* of the trajectory, τ is the length of the memory window, and *m* is the exponent in averaging; *m* = 3 for distances and *m* = −1 for angles, the latter corresponding to direct averaging.^11,14,15,58^ It should be noted that, except for small *t*, the normalization factor in eq 7 can be replaced with [τ (1 – e^–1^)]^−1^.

To evaluate the integral of eq 7, *Y*_*j*_(*t*), we use a recursive variant of the trapezoid formula (eq 8), which was also used in an earlier AMBER-package implementation of the time-averaged restraints.^58^ In our implementation, the integral is permanently updated every *n*_ave_ time steps with the simple average ⟨*Y*_*j*_⟩ over the time steps from *t*_*n*_ave_*I*+1_ to *t*_*n*_ave_(*I*+1)_, where *I* = *i* ÷ *n*_ave_ is the number of *n*_ave_-long sequences of MD steps until *t*_*i*_. Between *t* = *t*_*n*_ave_*I*+1_ and *t* = *t*_*n*_ave_(*I*+1)_, *Y*_*j*_ is computed using the momentary value of *y*_*j*_^–*m*^ (eqs 9 and 10). In this way, the target function always depends on the current molecular geometry and the restraint energy does not depend on trajectory history between *t* = *t*_*n*_ave_*I*+1_ and *t* = *t*_*n*_ave_(*I*+1)_, which assures total-energy conservation in this time period, should the simulations be run in the microcanonical mode. We demonstrate the advantage of this *n*_ave_-time-step update of the integral with respect to every-time-step update in section “Stability of Time-Averaged Simulations”.
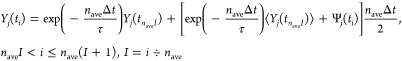
8
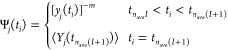
9
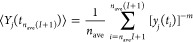
10

We also set
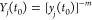
11

Finally, the *j*th average quantity at time step *t*_*i*_, , is calculated from the integral of the (−*m*)th power, *Y*_*j*_(*t*_*I*_), of the momentary observable *y*_*j*_(*t*) ≡ *y*_*j*_(**r**(*t*)) over the trajectory until the *t*_*i*_th time step (eq 12).
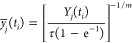
12

To calculate the time averages of the dihedral angles, their sines and cosines are time-averaged first and the average dihedral angles are calculated from these quantities by using the standard FORTRAN atan2 function.

The gradient due to time-averaged restraints is expressed by eq 13.
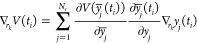
13where *V*(*t*_*i*_) is a shorthand for *V*(**r**(*t*_*i*_)), *N*_*r*_ is the total number of restraints of a given kind (distance or angular in this work), and
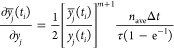
14

From eq 14 it follows that, for *t*_*i*_ ≫ τ, the derivatives of the time-averaged quantities and, thereby, the time-averaged-restraint gradients (eq 13) are scaled down by the factor of *n*_ave_Δ*t*/[τ(1 – e^–1^)]. The values of τ usually applied range from 5 to 50 ps.^14,15^ In this work we set τ from 4.89 to 489 ps (in UNRES MD, a “natural” time unit equal to about 48.9 fs is applied,^45^ hence we set τ to its integer multiplicity). Therefore, especially with *n*_ave_ = 1, the restraint contributions to forces are reduced to effectively turn the MD runs with time-averaged restraints into unrestrained runs. To ensure large enough force contributions from time-averaged restraints, we scale the time-averaged-restraint gradients by a factor *f*_*i*_ (at the *i*th time step) defined by eq 15.
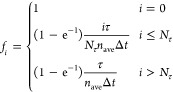
15where *N*_τ_ is the number of MD steps over which the scaling factor should be increased from 1 to (1 – e^–1^)τ/(*n*_ave_Δ*t*). This parameter is typically equal to the integer part of τ/Δ*t* but can be set by the user.

The gradual scaling of the restraint forces with the progress of an MD trajectory prevents us from overemphasizing the restraint contributions until enough MD steps have been executed to provide at least τ/(*n*_ave_Δ*t*) terms in the averaged quantities. On the other hand, we determined that the time-averaged-restraint components of the potential energy, which come into play when REMD/MREMD simulations are run, should not be scaled or the energy differences between replicas become too big for any replica exchange to happen. Moreover, postprocessing the REMD/MREMD simulations with WHAM to determine the weights of the conformations encounters insurmountable numerical problems due to big differences in the values of the scaled time-average-restraint energy components. We will demonstrate the advantages of the revised time-averaged-restraint algorithm proposed in this work in section “Essential Role of Restraint-Force Scaling in Time Averaged Simulations”.

### Systems Studied

**Figure 2:**
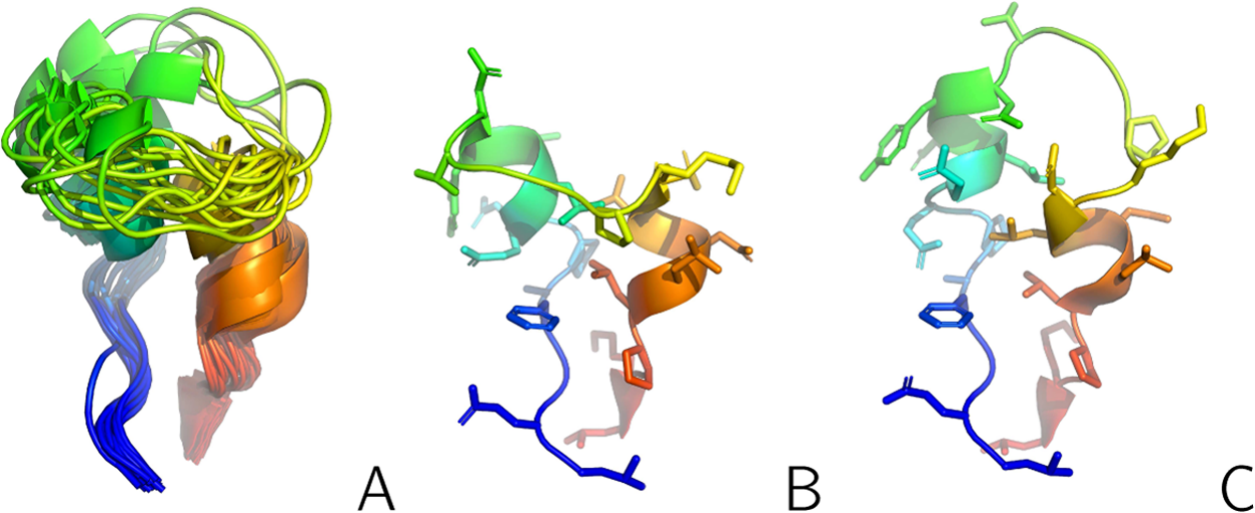
(A): The NMR-determined conformational ensemble of the L129–L153 fragment of 2KW5 from the respective PDB entry. (B, C): Structures #1 and #6 from this ensemble, which were used to calculate synthetic interproton-distance restraints. The backbone is shown in the cartoon representation, while the side chains in panels (B, C) are shown in the stick representation. The chains are colored from blue to red from the N- to the C-terminus. The side chains are omitted from panel (A). The drawings were made with PyMOL.^60^

To investigate the behavior of the UNRES MD/MREMD simulations with time-averaged restraints, we selected the L129–L153 loop part of the Slr1183 protein from *Synechocystis sp.* (PDB: 2KW5(59)). The respective conformational ensemble derived from the PDB: 2KW5 structure is shown in Figure 2A. It can be seen that the structure is largely disordered. For this system, we carried out calculations with synthetic interproton-distance restraints derived from structures #1 and #6 of the ensemble. These structures are shown in panels B and C of Figure 2. This system is hereafter referred to as 2KW5(129–153).

**Figure 3:**
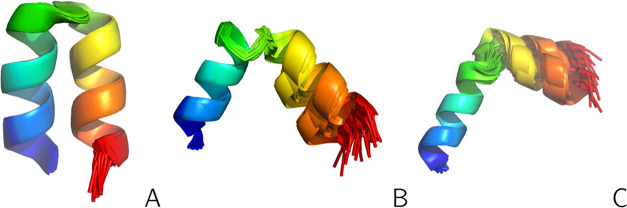
NMR-determined conformational ensembles of the three forms of 2LWA corresponding to structures A (panel A), B (panel B) and C (panel C)^61^ of the respective PDB entry. The backbones are shown in the cartoon representation and the side chains are omitted. The chains are colored from blue to red from the N- to the C-terminus. The drawings were made with PyMOL.^60^

To test the performance of UNRES with the time-averaged NMR-derived restraints feature with multistate proteins, we selected the influenza hemagglutinin fusion peptide (PDB: 2LWA,^61^ 25 residues). The NMR-determined conformational ensembles of three distinct forms of this protein (referred to in its PDB entry as chains A, B, and C, respectively, which are hereafter referred to as structures A, B, and C, respectively, in this paper), are shown in Figure 3A–C.

**Figure 4:**
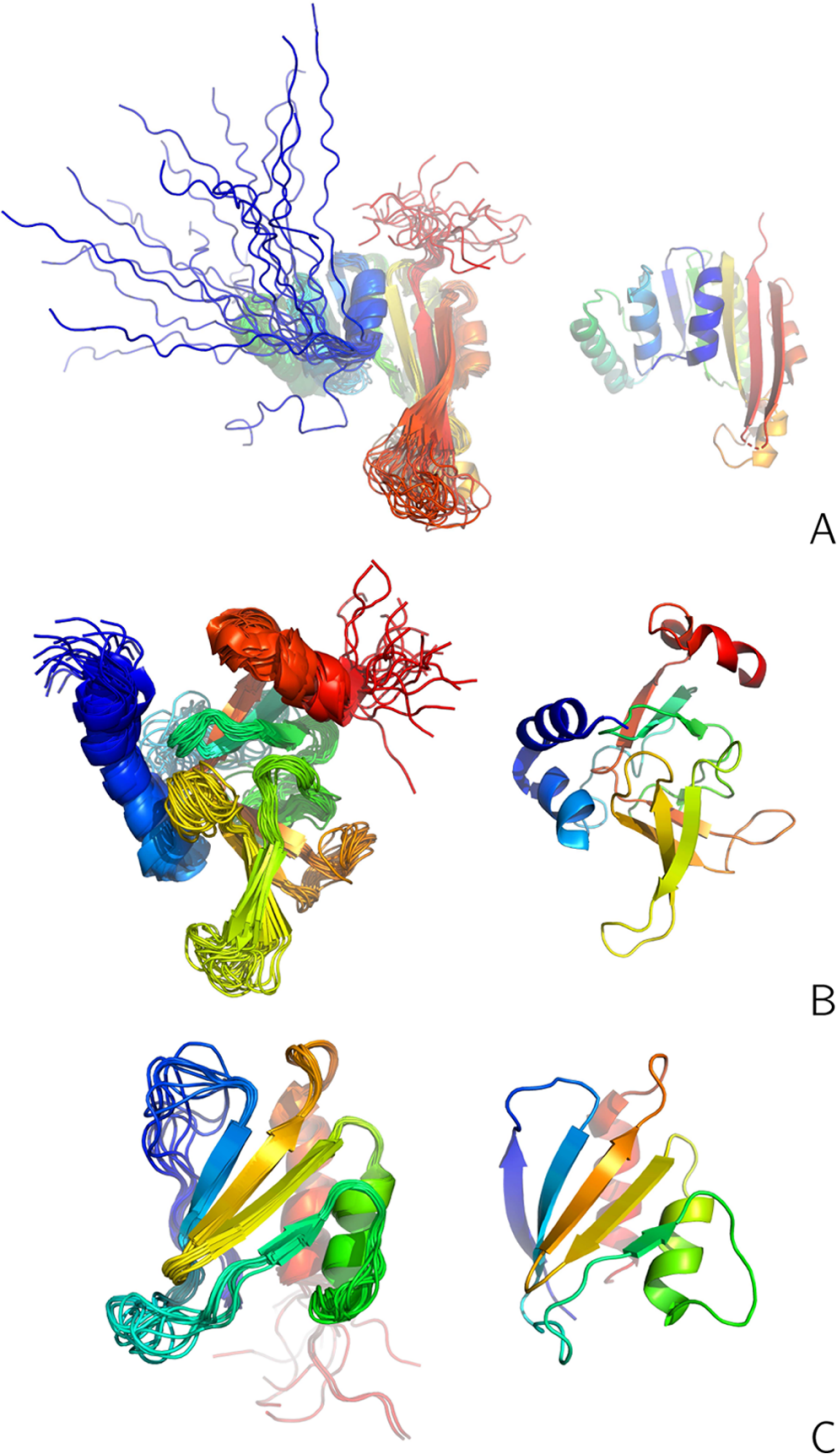
NMR-determined conformational ensembles (left) and the X-ray structures (right) of the three proteins of the Montelione/NEF Benchmark Data Set^62^ considered in this study: (A) 2KW5 (NMR)/3MER (X-ray); (B) 2KZN (NMR)/3E0O (X-ray); (C) 1PQX (NMR)/2FFM (X-ray). The backbones are shown in the cartoon representation, the chains colored from blue to red from the N- to the C-terminus. The drawings were made with PyMOL.^60^

To test our method with larger-size (partially disordered and not multistate) proteins, we selected two proteins from the Montelione/NEF Benchmark Data Set,^62^ for which both NMR and X-ray structures are available. These were the complete structure of 2KW5 (202 residues; its X-ray structure counterpart being 3MER), hereafter referred to as 2KW5 and the peptide methionine sulfoxide reductase msrB from *Bacillus subtilis* [PDB: 2KZN(59) (NMR), 3E0O (X-ray), 147 residues], hereafter referred to as 2KZN. For reference, we included one more protein from the Montelione/NEF Benchmark set, which has a well-defined structure, namely the *Staphylococcus aureus* protein SAV1430 [PDB: 1PQX (NMR), 2FFM (X-ray), 91 residues], hereafter referred to as 1PQX. The NMR structures of 2KW5 and 2KZN contain quite large disordered segments, which was reflected in the results of our earlier work,^53^ in which we found that the structures deposited in the PDB are consistent only with about 50% of experimental distance restraints. The NMR structure of 1PQX (which is well-defined) satisfies almost all of the experimental restraints. The NMR ensembles and the X-ray structures of these proteins are shown in Figure 4A–C. As in our previous work,^53^ we used the X-ray structures of these three proteins as reference structures.

### Calculation Procedure

Most of the canonical MD and MREMD simulations were carried out in the Langevin-dynamics mode (which also provides thermostatting), with the time step of Δ*t* = 4.89 fs. In some of the runs, which were aimed at checking the conservation of the bath temperature, the Berendsen thermostat^63^ was also used. The velocity-Verlet integrator^64^ adapted to the Langevin dynamics with UNRES,^34^ which uses the variable-time-step (VTS) algorithm^45^ was applied to solve the equations of motion. All simulations were carried out with the recently optimized UNRES code.^35^

All starting structures were random-generated by using the procedure described in ref (65). Briefly, a coarse-grained polypeptide chain is gradually built up starting from the N-terminus. To add the next residue, its backbone-virtual-bond-dihedral angle γ is sampled at random from the [−180°, 180°] interval, while its backbone-virtual-bond-angle θ as well as the zenith (α) and the azimuth (β) angles defining the local geometry of united side chain (Figure 1) are sampled from the Boltzmann distributions calculated from the respective knowledge-based potentials determined in ref (66). The Cartesian coordinates of the residue are calculated from the generated internal coordinates. Subsequently, its overlaps with the previous residues are checked and, if found, the generation is retried. If 100 retrials are unsuccessful, the generation is restarted from one more residue backward. For multitrajectory canonical simulations and MREMD simulations, different starting structures were generated for different trajectories.

For 2KW5(129–153), we ran both canonical and microcanonical simulations, the latter to determine the extent of energy conservation. Each series of canonical simulations was run at *T* = 300 K and consisted of 4 or 8 trajectories, 5,000,000 to 10,000,000 MD steps each.

For 2LWA, 2KW5, 2KZN, and 1PQX, MREMD simulations only were run at the following 12 temperatures: 260, 262, 266, 271, 276, 282, 288, 296, 304, 315, 333, and 370 K, respectively, which were selected by using the Hansmann algorithm^67^ to maximize the walks in the temperature space. Four replicas were run at a given temperature, resulting in a total of 48 replicas. Each replica consisted of 10,000,000 (2LWA) or 20,000,000 (the other proteins) time steps and the temperatures were exchanged between replicas every 10,000 time steps. The temperature was controlled by the Langevin thermostat, with scaling down the water friction by a factor of 0.05. The UNRES coordinates were saved every 10,000 time steps, i.e., every replica-exchange time. The last 1000 structures from each trajectory (48,000 structures total) were taken for further analysis.

The structures resulting from MREMD simulations were subjected to postprocessing with the UNRES implementation^49^ of the binless variant of WHAM^48^ to enable us to compute the statistical weights at the desired temperature within the replica-temperature range. Because the aim of time-averaged MREMD simulations is to produce equilibrium ensembles subject to time-averaged restraints, the time-averaged penalty terms are present in the extended effective energy expression (eq 1) which appear in the WHAM equations (eqs 14–16 in ref (49)). The part of an ensemble comprising 99% of conformations at a given temperature was subjected to a cluster analysis by means of the Ward minimum variance method.^68^ In this work, we collected ensembles at *T* = 280 K (for computing the ensemble-averaged distances and constructing the PDB-entry-style sets of NMR-determined conformations) or *T* = 260 K and *T* = 280 K for determining single conformations to compare with the X-ray structures. For comparison with the reference X-ray structures, the number of clusters (and, thereby, the number of models) was set at 5, this number being selected after the rules of Community Wide Experiments on the Critical Assessment of Techniques for Protein Structure Prediction (CASP),^69^ in which 5 models per target can be submitted for assessment. The families (and, consequently, the selected structures) were ranked by the cumulative probabilities of all conformations belonging to them, as described in our earlier work.^49^ The structure with the lowest restraint violation was selected as the representative of a given family. Additionally, the ensembles of 2LWA, 2KW5, 2KZN, and 1PQX were dissected into 20 families whose representative conformations best fitting the NMR data were selected to constitute reduced ensembles with a size typical of NMR-determined ensembles deposited in the PDB.

To compute the actual (not estimated with ESCASA) interproton distances and, subsequently, average interproton distances at the all-atom level, we ranked all structures of a given system according to decreasing weights determined by WHAM, given *T* = 280 K, and took the ensemble composed of those whose sum of weights was 0.99. These structures were subsequently converted to all-atom representation by using the cg2all algorithm,^70,71^ followed by refinement with AMBER^72^ with the ff19SB force field^73^ and implicit-solvent Generalized Born Surface Area (GBSA) model,^74,75^ as described in our earlier work.^56^ Then we computed the interproton distances for all structures. Finally, we computed the *r*^–6^-averaged distances using eq 16.
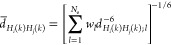
16where *d*_*H*_*i*_(*k*)*H*_*j*_(*k*)_ is the average distance between the protons that belong to restraint *k*, *N*_e_ is the number of structures in the ensemble, *w*_*l*_ is the weight of the *l*th conformation of the ensemble (determined by WHAM; the weights are normalized to 1), and *d*_*H*_*i*_(*k*)*H*_*j*_(*k*);*l*_ is the interproton distance for the *l*th conformation. If the restraint corresponds to an average over equivalent proton groups (such as, e.g., the H^β^ protons of isoleucine or the H^δ^ protons of phenylalanine), all distances between the individual protons of these groups are *r*^–6^-averaged for a given conformations, with equal weights.

The same procedure was applied to the reduced ensembles of conformations of 2LWA, 2KW5, 2KZN, and 1PQX (for which the weights were summed over each cluster to obtain the weight of the representative of a given family representative) and to the PDB ensembles (for which the weights of all conformations were equal).

The distance-boundary violations were quantified as the right upper distance boundary root-mean-square deviations (ρ_*u*_^+^) defined by eq 17, the total number of interproton distances greater than the respective upper distance boundary, and the number of distances greater by 2 Å or more than the upper distance boundaries.
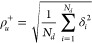
17
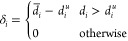
18where *N_d_* is the number of interproton-distance restraints, *d*_*i*_ is the ensemble-averaged (cf. eq 16) distance from the simulated or PDB ensemble and *d*_*i*_^*u*^ is the upper distance boundary.

As measures of fitting the simulated structures to the reference structures, we used the root-mean-square deviation of the C^α^-atom positions (C^α^-RMSD or, in short, RMSD) at the optimal superposition of the C^α^ atoms of the target structure on those of the reference structure and the Global Distance Test Test Score (GDT_TS).^76,77^ The latter is an average of the percentages of the target structure whose C^α^ atoms superpose within 1, 2, 4, and 8 Å, respectively, on those of the reference structure. The TMScore software from Zhang’s lab^78^ was used to calculate both measures.

### Experimental and Synthetic Restrains

The experimental restraints were the version-2 (v.2) restraints taken directly from the PDB entries and converted to the UNRES input format. The distance restraints pertaining to the same residue were omitted because they do not contribute useful information at the coarse-grained level.

For testing the method with the 2KW5(129–153) system, we generated interproton-distance restraints from conformation #1 (Figure 2B; a total of 173 restraints) and conformation #6 (Figure 2C; a total of 159 restraints), as well as *r*^–6^-averaged distance restraints from both structures, calculated by using eq 16 with two weights only, each one equal to 0.5, of its PDB ensemble (a total of 186 restraints). These distance restraints are collected in the respective machine-readable files of the Suppdata.zip archive of the Supporting Information (see section “Glossary of the machine-readable files” of the Supporting Information for detailed content of the archive).

The experimental distance and angular restraints (both the original restraints on the ϕ and ψ angles and those on the θ and γ angles after transformation to the coarse-grained representation, which were used in the actual calculations) for 2LWA, 2KW5, 2KZN, and 1PQX are collected in the respective machine-readable files of the Suppdata.zip archive of the Supporting Information and the numbers of these restraints are collected in Table 1. Although both distance and angular restraints were used in the calculations, in what follows we discuss only the violations of the distance restraints. The reason for this is that even if the angular restraints on the coarse-grained geometry are fulfilled, the original restraints on the ϕ and ψ angles (the values of which are calculated after converting the coarse-grained structures to the all-atom structures) are often violated. Therefore, restraints should be imposed on the ϕ and ψ angles estimated analytically from the coarse-grained geometry in a similar way as the estimation of proton positions in the ESCASA approach.^40^ In this work, we treat the restraints on the θ and γ angles derived from those on the ϕ and ψ angles only as a crude way to keep control over the backbone-local geometry.

**Table 1:** Numbers of NMR-derived Restraints on Interproton Distances, Backbone-virtual-bond-dihedral Angles γ, and Backbone-virtual-bond Angles θ Used in NMR-data-assisted UNRES/MREMD Simulations

	# restraints on		
protein	distances	γ	θ
2LWA	175	18	20
2KW5	947	134	147
2KZN	578	104	121
1PQX	1015	64	74

## Results and Discussion

### Stability of Time-Averaged Simulations

**Figure 5:**
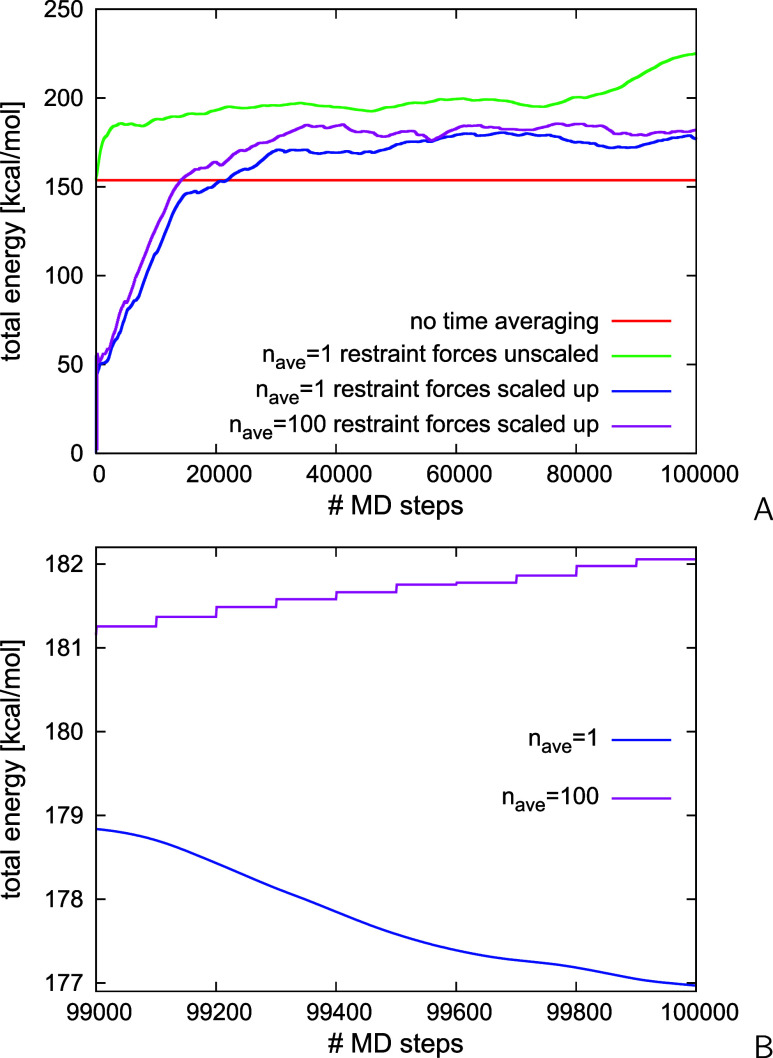
(A) Variation of the total energy in the restrained NVE MD (with a small time step of Δ*t* = 0.489 fs) runs of 2KW5(129–153) without time averaging (run 1) and with time averaging (τ = 4.89 ps): no force scaling, *n*_ave_ = 1 (run 2), force scaling up by (1 – e^–1^)τ/Δ*t*: *n*_ave_ = 1 (run 3), and *n*_ave_ = 100 (run 4). (B) A close-up of run 3 and run 4 for the last 1000 time steps. In runs 3 and 4, the restraint energy was scaled up along with the restraint forces to check total-energy conservation. The plots were made with gnuplot.^79^

Because the potential-energy function in time-averaged-restraint simulations contains a time-dependent term (eq 7), the total energy is not conserved in the microcanonical mode. To determine the extent of energy nonconservation in time-averaged-restraint simulations, we carried out MD runs in the microcanonical (NVE) mode (constant number of particles, volume and total energy) for the 2KW5(129–153) system with the synthetic distance restraints generated from conformations #1 and #6 of its NMR ensemble (see the Supporting Information for the name and location of the restrain files) without (run 1) and with time averaging (runs 2–4), respectively. For each run, 100,000 MD steps were executed with a small time step of Δ*t* = 0.489 fs, the initial velocities corresponding to the temperature of 300 K (however, the temperature is not conserved in NVE runs). The value of τ in eq 7 in runs 2–4 was 4.89 ps and the value of *n*_ave_ was 1 in runs 2 and 3 and 100 in run 4. The restraint-potential well-depth (*A* in eq 2) was 5 kcal/mol. In run 2, the restraint forces were not scaled, while in runs 3 and 4 they were scaled up by the factor of (1 – e^–1^)τ/(*n*_ave_Δ*t*) (cf. section “Time-Averaged Restraints”). The plots of the total energy vs time are shown in Figure 5A,B. It can be seen from Figure 5A that the total energy remains effectively constant (the oscillations not exceeding 0.001 kcal/mol) in restrained microcanonical MD simulations without time averaging (run 1). In all time-averaged simulations (runs 2–4), the total energy is not constant even when the time-averaged restraint forces are not scaled, which introduces only a small perturbation. The initial total-energy drift observed in the plots arises because the 4.89 ps memory window is sufficiently filled only after about 10,000 MD steps, during which the averaged restraints are being built, but the total energy continues to vary after this. The average-update frequency (*n*_ave_) does not have a major effect on the total-energy variation in a long run. However, as can be seen from Figure 5B, in which the total energy is plotted for the last 1000 steps of runs 3 and 4, the total energy stays constant in the *n*_ave_-step-long intervals.

Nonconservation of the total energy in the microcanonical mode could result in a substantial increase of the kinetic temperature in the canonical mode and, thereby, in significant errors in the generated conformational ensembles. To determine the extent of the problem, we carried out a series of canonical (NVT) runs for the 2KW5(129–153) system (with restraint-potential well-depth *A* = 5 kcal/mol), each consisting of 4 independent 10,000,000-step trajectories with the time step of Δ*t* = 4.89 fs (which is the time step used in the production simulations), without time averaging and with different variants of time averaging. The temperature was controlled by the Berendsen^63^ or the Langevin thermostat, the latter with scaling the water friction by a factor of 0.02 or 0.05, respectively. The thermal-bath temperature was set at *T*_bath_ = 300 K. The average kinetic temperature was calculated from the second half of each of the 4 trajectories of a given run. The average temperatures, along with run settings, are collected in Table 2. It can be seen that the average kinetic temperature does not differ much from that of the thermal bath for the runs without time averaging but it is significantly higher in the time-averaged-restraint runs for the smallest value of τ = 4.89 ps, the difference decreasing with increasing *n*_ave_. With the highest τ = 489 ps and with *n*_ave_ = 1000, the average temperatures approach those obtained for the runs without time averaging. The temperature is the most close to the bath temperature for the Berendsen thermostat; however, using this thermostat results in a very narrow kinetic-energy distribution. Therefore, we selected the Langevin thermostat with water-friction scaling of 0.05, even though it results in a slightly slower dynamics compared to that with the scaling factor of 0.02. Based on the obtained results, we used τ = 48.9 ps and *n*_ave_ = 500 or τ = 489 ps and *n*_ave_ = 1000 in most of the calculations. We also noted that temperature conservation becomes better as the system size increases.

**Table 2:** Average Kinetic Temperature from the Series of 4-Trajectory MD Runs (10,000,000 Steps with a 4.89 fs Time Step Per Trajectory) without Time Averaging and with Time Averaging with Different Values of τ and *n*_ave_

		*T*_kin_ [K]		
τ [ps]	*n*_ave_	Ber.	Lang. 0.02	Lang. 0.05
No Time Averaging				
		300.0	301.4	303.1

Time Averaging				
4.89	1	327.2	542.1	365.2
	10	326.6	532.1	362.1
	100	316.0	425.9	336.2
48.9	1	306.0	346.0	318.4
	10	306.0	345.1	318.4
	100	305.4	341.1	317.2
	500	303.5	328.2	312.7
	1000	302.2	318.9	309.4
489.0	1000	300.5	305.3	304.6

### Essential Role of Restraint-Force Scaling in Time Averaged Simulations

To demonstrate the necessity of restraint-force scaling in restrained MD simulations with time averaging, we carried out 4 series of canonical-MD runs with 2KW5(129–153). Each run consisted of 4 trajectories, 1,000,000 steps each with a 4.89 fs time step, at *T* = 300 K. It can be seen from Table 2 that the kinetic temperature is for this system by about 36 deg higher than the bath temperature when the time-averaged-restrain forces are scaled. The rationale of setting a relatively small τ was to check how does the method perform at the boundary of stability limit. Runs 1–3 were carried out with restraints derived from structure #1. Run 1 was carried out without time averaging, while runs 2 and 3 were carried out in the time-averaged mode with τ = 4.89 ps, with (run 2) and without (run 3) restraint-force scaling, respectively. Finally, run 4 was carried out without any restraints.

**Figure 6:**
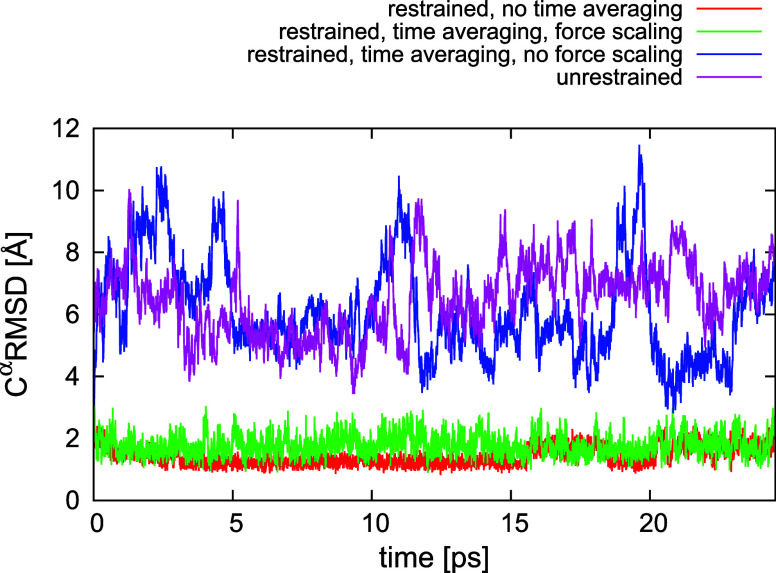
Superposed plots of the variation of C^α^-RMSD of the conformations of 2KW5(129–153) from structure #1 obtained in restrained canonical MD simulations with UNRES (restraint well depth *A* = 5 kcal/mol) with interproton-distance restraints calculated from structure #1 without time averaging (run 1), with time averaging (τ = 4.89 ps, *n*_ave_ = 10) and restraint-force scaling (run 2), with time averaging and no restraint-force scaling (run 3), and in an unrestrained MD run (run 4). The plot was made with gnuplot.^79^

The plots of the C^α^-RMSD from structure #1 are shown in Figure 6. As can be seen, low RMSD has been obtained in the restrained simulations without time averaging. This result could be expected because the restraints were derived from a single conformation. The simulation with time averaging and without restraint-force scaling resulted in high RMSD values given the small size of the system considered here, the RMSD range being not much different from that from unrestrained simulations. Only with restraint-force scaling did the RMSD values become comparable to those from regular restrained simulations.

It should be noted that the kinetic temperature for the run with unscaled time-averaged restraint forces is 303.3 K, which means that the high RMSD obtained in this run could not result from the elevated kinetic temperature but only from not scaling the restraints. As pointed out above, the kinetic temperature was remarkably elevated with scaled restraint forces which, however, did not prevent the simulation from reaching conformations close to the reference structure. This result demonstrates the robustness of the method even when the time window is short, which results in a remarkably elevated kinetic temperature with respect to the bath temperature.

### Effect of Restraint Averaging on Simulated Structures

In this part of our study, we carried out calculations with synthetic restraints derived from structure #1, #6, or both, of 2KW5(129–153) (Figure 2B,C), in order to determine the behavior of the method when the reference structure(s), which the restraints correspond to are known.

**Figure 7:**
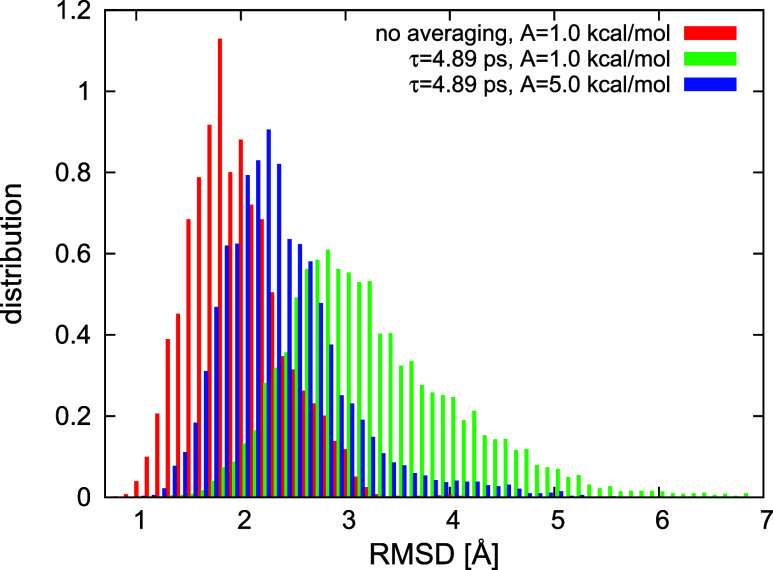
C^α^-RMSD distributions from the NMR structure #1 of the 2KW5(129–153) conformations obtained in canonical MD simulations with interproton-distance restraints derived from structure #1 without time averaging and with time averaging with the restraint-well depth of 1.0 and 5.0 kcal/mol, respectively. See text for the other settings of the simulations. The plots were made with gnuplot.^79^

First, we determined the effect of time averaging on the results of the simulations with the restraints derived from one well-defined structure. To accomplish this task, we carried out canonical MD simulations of 2KW5(129–153) with synthetic interproton-distance restraints derived from structure #1. We carried out one series of runs with nonaveraged restraints, with the restraint-potential well-depth *A* = 1 kcal/mol, and two series of runs with time-averaged restraints, with τ = 4.89 ps, *n*_ave_ = 100 and *A* = 1 or 5 kcal/mol, respectively. The relatively small τ was set to determine if the method can produce conformational ensembles compatible with the restraints even with a small memory-window length. Each series consisted of 4 trajectories of 10,000,000 MD steps with a 4.89 fs step size. The plots of the RMSD distribution functions collected from the last 2,000,000 steps of all trajectories of a given series are shown in Figure 7. As shown, introducing time-averaged restraints results in right-shifting and broadening the RMSD distribution, increasing the restraint well-depth to 5 kcal/mol resulting in a smaller shift. Increasing *A* beyond the value of 5 kcal/mol reduces the shift but we found that already the value of 10 kcal/mol could cause instability due to excessive time-average-restraint forces. Consequently, the value of 5 kcal/mol is a reasonable compromise between approaching the results of restrained simulations without time averaging for nonaveraged restraints and numerical stability. The shift results from time averaging, owing to which the distances are averaged over a time window and, consequently, a single conformation does not need to satisfy all restraints. Therefore, the obtained ensemble is more diffuse compared to that resulting from simulations without time averaging. Because the RMSD range has the left boundary of 0, the distribution is asymmetric, as can be seen from Figure 7. Consequently, increasing the spread of the conformational ensemble not only makes the RMSD distribution broader but also right-shifted.

Subsequently, we carried out the canonical MD simulations for the 2KW5(129–153) system with the synthetic restraints derived from structures #1 and #6, without (run series 1) and with (run series 2) time averaging, respectively. For reference, we carried out two series of runs without and with time averaging with restraints derived from structure #1 (run series 3 and 4) and two series of runs with restraints derived from NMR structure #6 only (run series 5 and 6). Each run series consisted of 4 trajectories, 10,000,000 4.89 fs time steps each, with the restraint-potential-well depth *A* = 5 kcal/mol.

**Figure 8:**
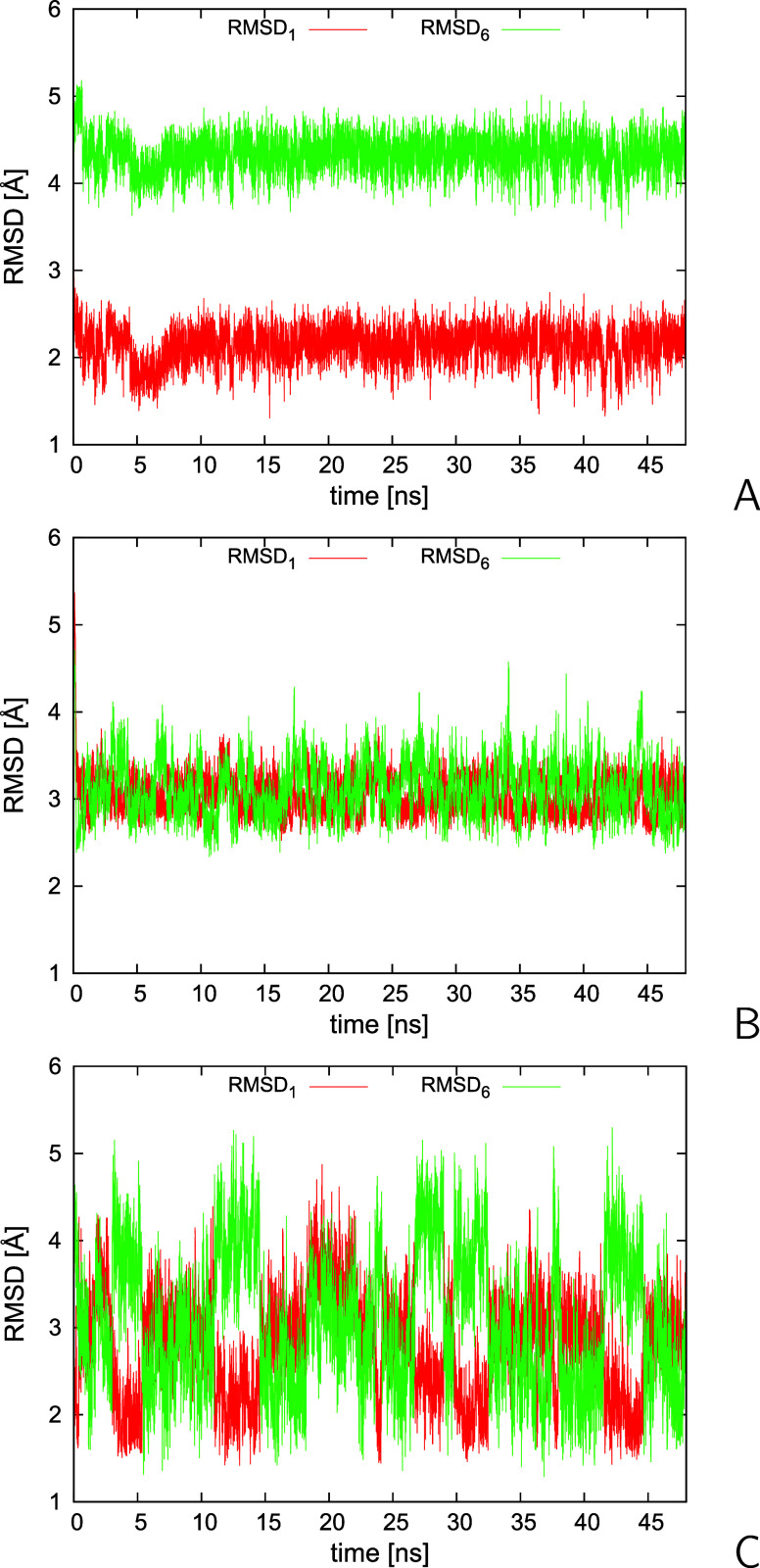
Variation of C^α^-RMSD from the PDB-ensemble structures #1 (RMSD_1_) and #6 (RMSD_6_) of the 2KW5(129–153) system with simulation time in sample trajectories of canonical MD simulations with synthetic average interproton-distance restraints derived from structures #1 and #6. (A, B) Simulations without time averaging. (C) Simulations with time averaging (τ = 4.89 ps, *n*_ave_ = 10). The plots were made with gnuplot.^79^

The variation of C^α^-RMSD from structures #1 and #6 for two sample trajectories from run series 1 is shown in Figure 8A,B and that for a sample trajectory from run series 2 is shown in Figure 8C, respectively. The other trajectories exhibit similar patterns of RMSD variation. As can be seen, without time averaging, the resulting structures are either stuck in the neighborhood of structure #1 (low RMSD from structure #1 and high from structure #6; Figure 8A) or in conformations with a moderate distance from structure #1 and structure #6 (Figure 8B). Conversely, with time averaging, the system alternates between the two parent structures (Figure 8C).

**Figure 9:**
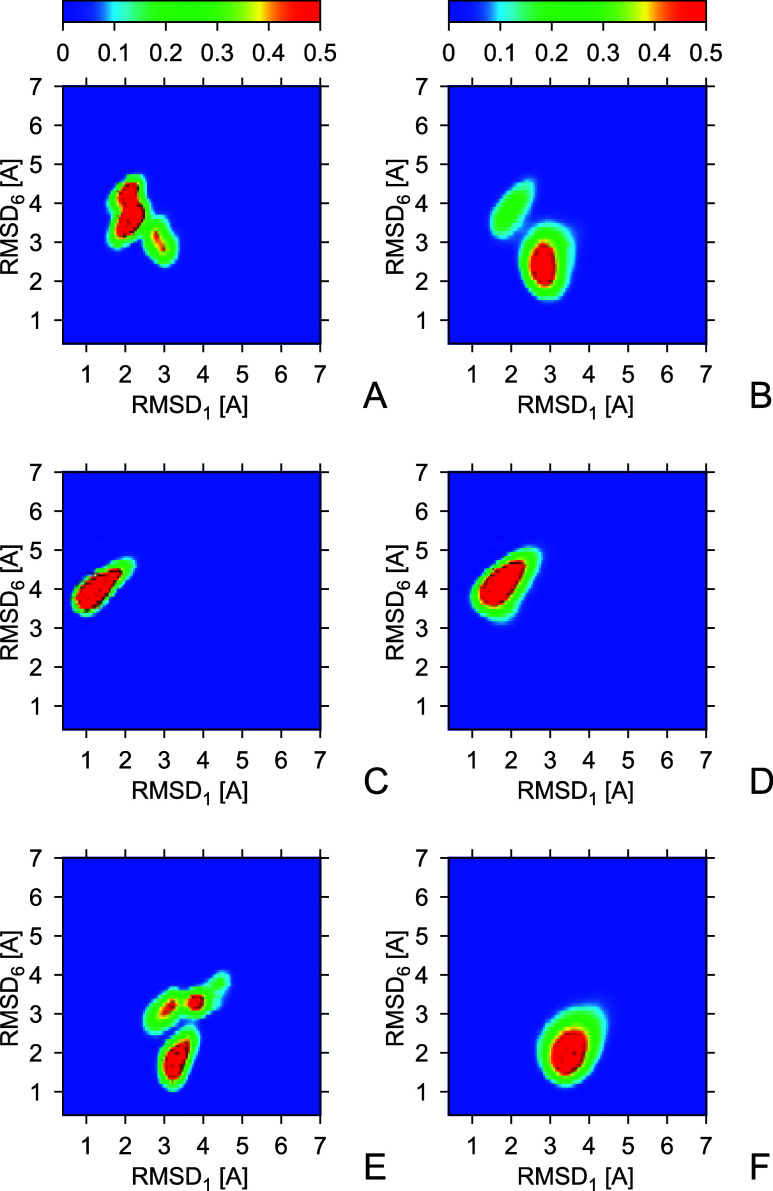
Two-dimensional maps of the C^α^-RMSD distributions from structure #1 (RMSD_1_) and #6 (RMSD_6_) calculated from the 2KW5(129–153) conformations obtained in canonical MD simulations with interproton-distance restraints derived from structures #1 or #6 or both. (A) Simulations without time averaging, restraints from structures #1 and #6. (B) Simulations with time averaging, restraints from structures #1 and #6. (C) Simulations without time averaging, restraints from structure #1. (D) Simulations with time averaging, restraints from structure #1. (E) Simulations without time averaging, restraints from structure #6. (F) Simulations with time averaging, restraints from structure #6. See text for the other settings of the simulations. The color scale of probability density is above the graphs. The plots were made with GRI.^80^

The two-dimensional probability-distribution maps in the RMSDs from NMR structures #1 and #6, respectively (RMSD_1_ and RMSD_6_, respectively) for all six run series are shown in Figure 9A–F. It can be seen that the RMSD distribution obtained from run series 1 (Figure 9A) indicates that the obtained ensemble is closest to NMR structure #1, with only a minor part of structures being kind of similar to structure #6. Conversely, with time averaging (run series 2), there are two clear lobes, one corresponding to conformations closer to structure #1 and the other one to structure #6, respectively (Figure 9B). The distributions from run series 3 and 4 (Figure 9C,D) demonstrate that the restraints from structure #1 exclusively result in conformations closer to structure #1 than those obtained with restraints averaged over structures #1 and #6. This observation pertains to the structures obtained both with and without time averaging, the RMSD being lower for the run series without time averaging, as already observed in Figure 7. A similar conclusion regarding the similarity to structure #6 can be drawn by comparing the distributions from run series 2 (Figure 9B) and run series 5 and 6 (Figure 9E,F). However, with the restraints from structure #6 and without time averaging, the RMSD distribution has 3 lobes, only one of which corresponds to conformations close to structure #6 (Figure 9E). Conversely, with time averaging, the RMSD distribution is unimodal, with the maximum corresponding to conformations close to structure #6 and far from structure #1. Consequently, while time averaging shifts the distribution of conformations slightly farther away from the reference structure, it seems to improve the ergodicity of simulations.

### Test with a Small Multistate Protein (2LWA)

To test the performance of the data-assisted protein-structure modeling with UNRES and time-averaged restraints, we selected the 2LWA protein. The original paper on its NMR structure determination with the aid of Xplor-NIH^61^ reports three families of solution conformations: a helical hairpin (structure A of the 2LWA PDB entry), a partially open helical hairpin (structure B) and a kinked helix (structure C), as shown in Figure 3. We ran the NMR-assisted UNRES/MREMD simulations at 12 temperatures, each quadruplexed (48 replicas total), the temperatures distributed as described in section “Calculation Procedure”. We implemented both the distance restraints (with the restraint-well depth *A* = 5 kcal/mol; eq 2) and the restraints on the backbone-virtual-bond angles θ (eq 3) and backbone-virtual-bond-dihedral angles γ (eq 4) calculated from the original restraints on the ϕ and ψ backbone-dihedral angles. The time-averaged simulations were carried out with τ = 48.9 ps, *n*_ave_ = 500 and τ = 489 ps, *n*_ave_ = 1000. The latter gave slightly better results in terms of fitting the calculated ensemble-averaged interproton distances to the NMR data.

**Figure 10:**
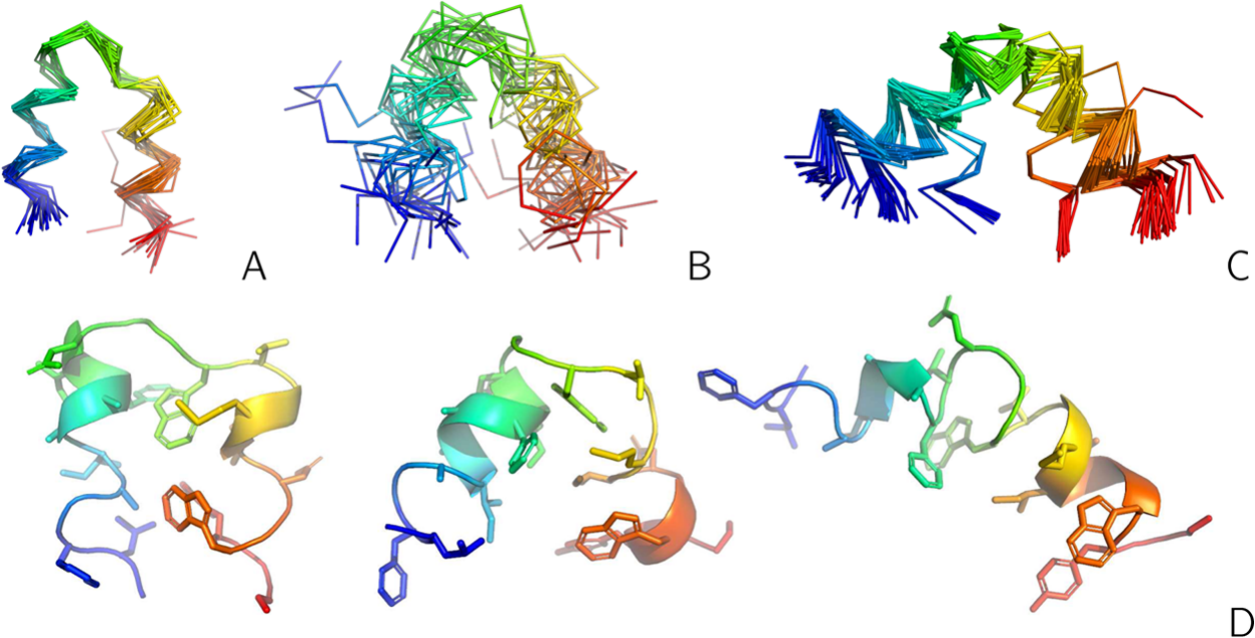
(A) Superposition of the C^α^ traces of the representative conformations of 20 families of 2LWA obtained in NMR-data-assisted UNRES/MREMD simulations without time averaging. (B) As in (A) but with time averaging (τ = 489 ps, *n*_ave_ = 1000). (C) Superposition of the C^α^ traces of all conformations of structures A, B, and C from the 2LWA PDB entry. (D) Three representative conformations of the ensemble shown in panel (B), which are closest to structure A, structure B, and structure C of the 2LWA PDB entry, respectively, shown in ribbon (for backbone) and stick (for side chains) representations. The chains are colored from blue to red from the N- to the C-terminus in all panels. The drawings were made with PyMOL.^60^

Subsequently, for each run, we constructed a representative subensemble of 20 conformations, as described in section “Calculation Procedure”. The PDB files of these ensembles are included in the Suppinfo.zip archive of the Supporting Information. The ensembles obtained without and with time averaging, with τ = 489 ps, *n*_ave_ = 1000, are shown in the C^α^-trace representation in Figure 10A,B, respectively. For reference, the ensemble composed of all superposed structures from the 2LWA PDB entry is shown in Figure 10C. It can be seen that all structures obtained without time averaging are helical hairpins forming a tight bundle and are thus similar to the 2LWA structure A but not to structures B and C deposited in the PDB (cf. Figure 3A). With time averaging, the obtained structures correspond to helical hairpins with the gradually increasing interhelix angle (Figure 10B). This feature of the obtained ensemble is emphasized in panel D of the Figure, in which three representative conformations are shown in the cartoon (backbone) and stick (side chains) representation. These structures are similar to structures A, B, and C, respectively, of the 2LWA PDB entry. Compared to the ensemble of 2LWA obtained with time averaging (Figure 10B), which consists of a continuity of conformations, the compact conformations of structure A from the 2LWA PDB entry are clearly distinguished from those of structures B and C.

**Figure 11:**
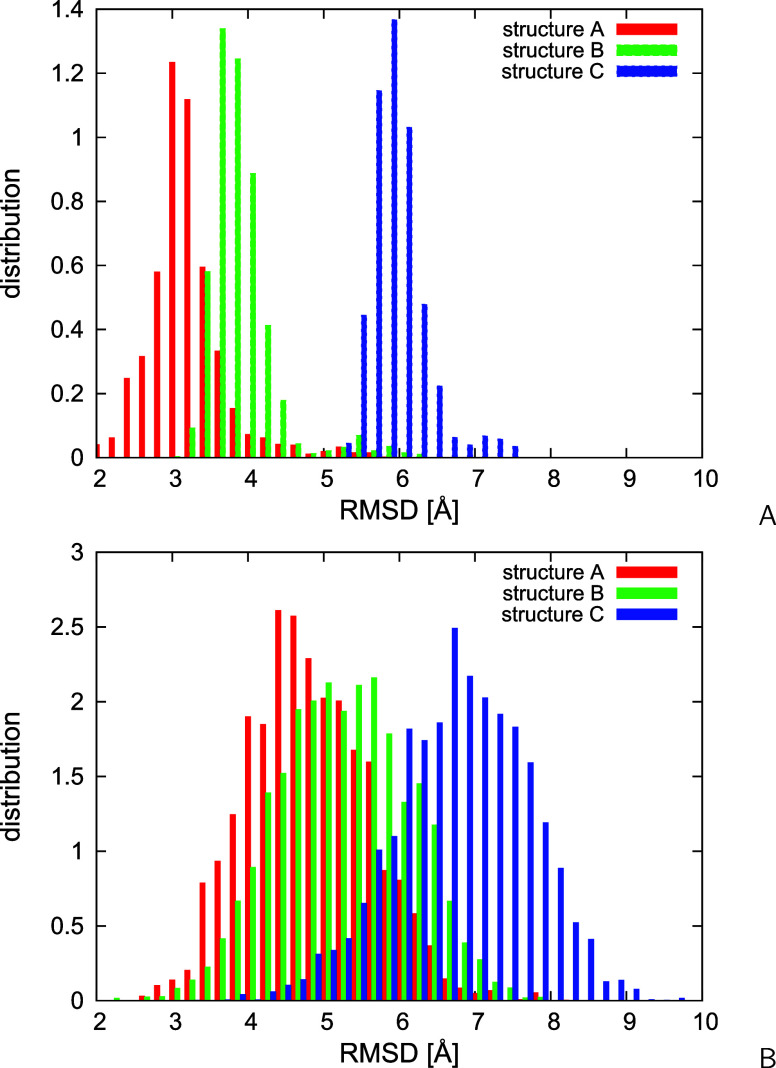
Distributions of C^α^-RMSD of the structures of 2LWA from PDB structures A, B, and C for the conformational ensembles obtained in NMR-data-assisted MREMD simulations (A) without time averaging and (B) with time averaging. The plots were made with gnuplot.^79^

The differences between the ensembles resulting from the two modes of restrained simulations are also illustrated in Figure 11A,B, where the C^α^-RMSD distributions from the PDB structures A, B, and C are plotted. As can be seen from Figure 11A, narrow RMSD distributions are obtained without time averaging and that corresponding to structure A is remarkably shifted to the left. The lowest RMSD from structure B is 3.2 Å and that from structure C is 5.6 Å. This feature is consistent with the presence of helical-hairpin conformations only in the reduced ensemble of 20 representative conformations, which are most similar to PDB structure A (Figure 10A). With time averaging (τ = 489 ps), the RMSD distribution from structure A becomes broader, shifts to higher values, and largely overlaps with the RMSD distribution from structure B (Figure 11B), which is consistent with the presence of a continuity of structures between a helical hairpin and a partially open helical hairpin. It can also be seen that the RMSD from the kinked-helix structure C decreases, consistent with the presence of a kinked-helix structure in the reduced ensemble of 20 representative conformations (Figure 10D).

**Figure 12:**
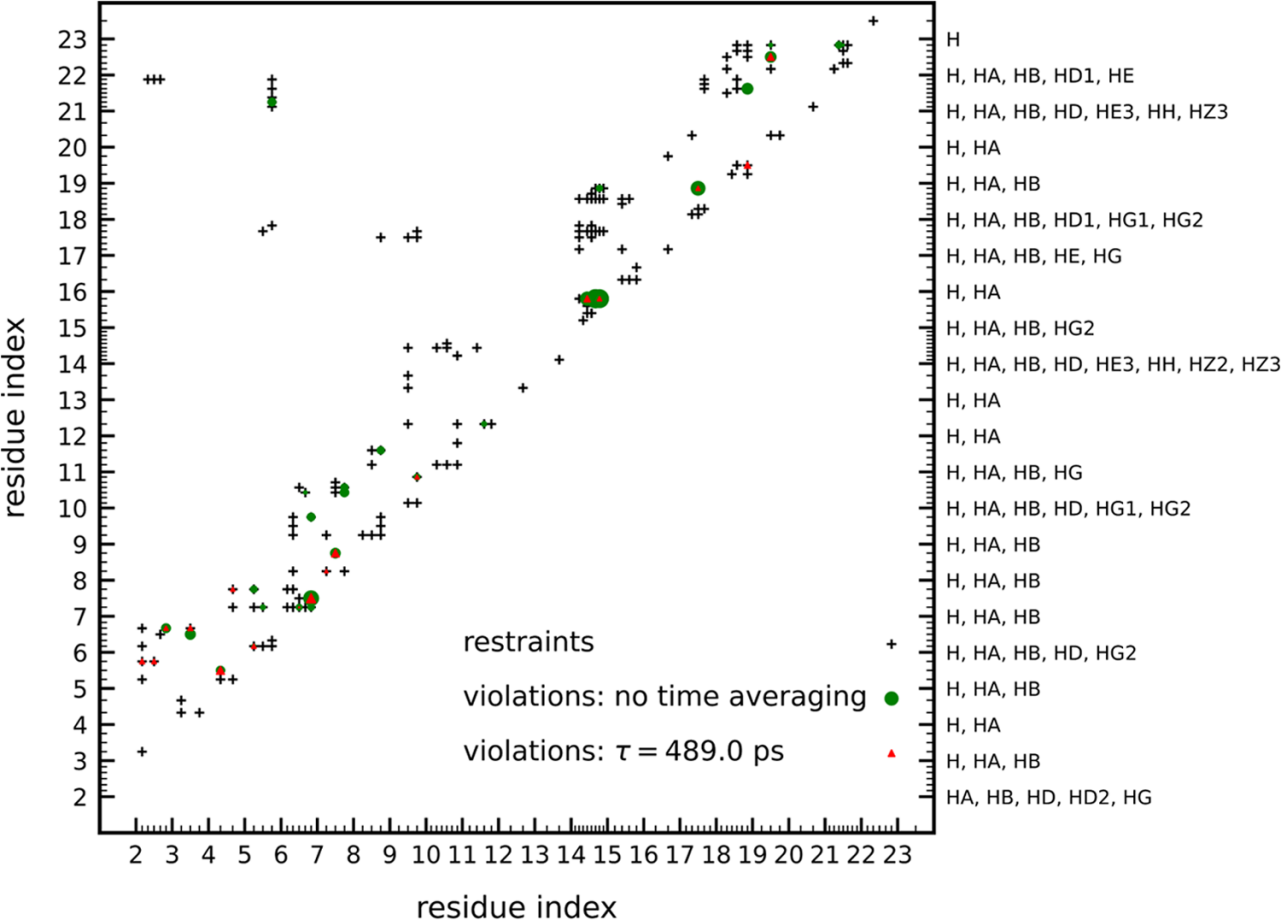
A diagram of NMR-determined interproton-distance restraints (black crosses) and violations of the upper boundaries of these restraints by ensemble-averaged interproton distances from NMR-assisted modeling of 2LWA with UNRES/MREMD (colored symbols). The size of the respective symbol is proportional to the deviation of the ensemble-averaged distance from the upper distance boundary. Green circles: simulations without time averaging. Red triangles: simulations with time averaging (τ = 489 ps, *n*_ave_ = 1000). The size of a symbol is proportional to the extent of upper-boundary violation. Each small tick corresponds to a proton of the residue with index below (ordinates) or to the left (abscissa) of it. The respective proton labels are shown on the right. The interproton distances of the consecutive conformations of the respective ensemble were calculated after conversion to all-atom structures.

**Figure 13:**
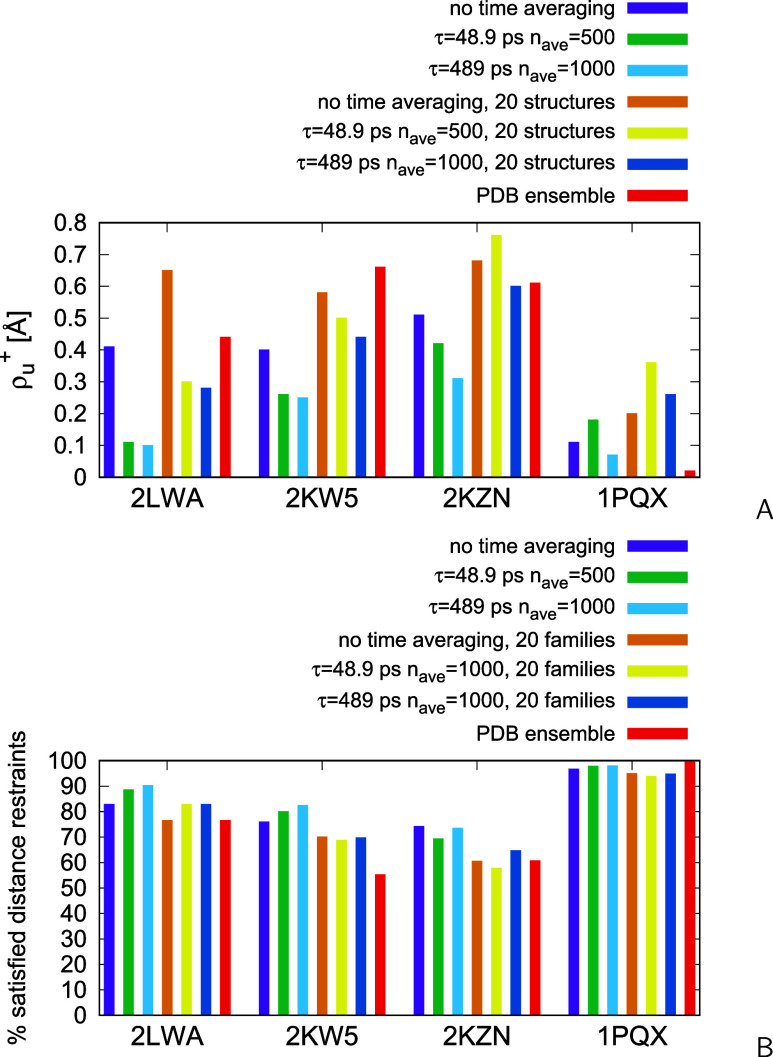
(A) Bar plot of the ρ_*u*_^+^ values of ensemble-averaged interproton distances from the right distance boundaries (eq 17). (B) Bar plot of the percentages of satisfied interproton-distance restraints for 2LWA, 1PQX, 2KW5, and 2KZN calculated from the results of MREMD simulations without time averaging and with time-averaged restraints, with τ = 48.9 ps, *n*_ave_ = 500 and τ = 489 ps, *n*_ave_ = 1000, respectively. The plots were made with gnuplot.^79^

The interproton-distance restraints and restraint violations without time averaging and with time averaging for τ = 489 ps, *n*_ave_ = 1000 (calculated after the conversion of the coarse-grained structures to all-atom structures with cg2all^70,71^) are shown in Figure 12. The numerical values of the differences of the specific interproton distances from the upper distance boundaries from all runs are included in the Suppdata.zip archive of the Supporting Information. The ensemble-averaged right RMSDs from the upper interproton-distance boundaries, (ρ_*u*_^+^s; eq 17) and the percentages of satisfied distance restraints, calculated from all-atom structures for all runs, and from the PDB ensemble, are shown as bar plots in Figure 13A,B, respectively. The corresponding numerical values, and the values obtained from interproton distances estimated by ESCASA are collected in Table S1 of the Supporting Information.

The ensemble-averaged ρ_*u*_^+^s calculated from the all-atom structures obtained by backmapping with cg2all,^70,71^ are 0.41 and 0.10 Å without and with time averaging (τ = 489 ps), respectively, and the numbers of violated restraints are 30 and 17, respectively; additionally the distance boundaries of 2 restraints are violated by more than 2 Å for the calculations run without time averaging. The ρ_*u*_^+^ values computed from the interproton distances estimated with ESCASA are 0.49 and 0.21 Å for the calculations without and with time averaging, respectively, and the numbers of distance-boundary violations are 50 (3 exceeding 2 Å) and 28 (none exceeding 2 Å), respectively. The ρ_*u*_^+^ calculated from the 2LWA PDB ensemble (a total of 60 structures) is 0.44 Å, with 41 violated distance restraints and 1 restraint violated beyond 2 Å. These violations are significantly larger than those obtained from our time-average-restraint data-assisted modeling with UNRES. Moreover, the ρ_*u*_^+^, the total number of violated distance restraints, and the number of restraints violated beyond 2 Å calculated from the subensemble of 20 structures obtained by clustering the ensemble obtained in this work with time averaging are 0.28 Å, 30, and 1, respectively, being still lower than those calculated from the conformations of the 2LWA PDB structure (Figure 13 and Table S1 of the Supporting Information).

### Tests with Larger Proteins with Disordered Regions

In this part of our work, we carried out MREMD simulations of three proteins for which both NMR and X-ray structures are available: 2KW5 (3MER), 2KZN (3E0O), and 1PQX (2FFM) (cf. section “Systems Studied”). These proteins were part of the test set used in our previous work^53^ to evaluate the performance of NMR-data-assisted UNRES simulations with interproton distances estimated using ESCASA.^40^ For each of the three proteins, we carried out an MREMD simulation without time averaging and two simulations with time averaging, one with τ = 48.9 ps, *n*_ave_ = 500, and another one with τ = 489 ps, *n*_ave_ = 1000, respectively. In our previous work,^53^ Hamiltonian replica exchange molecular dynamics (HREMD) simulations for these proteins were carried out. In HREMD, alteration of the energy function is another, apart from temperature, dimension of the replicas. Typically, the alteration is done by varying the weight of one or more of the energy components. Each of the temperatures is combined with each of the weights, making a 2D grid of replicas. In the HREMD simulations of ref (53), the restraint-penalty terms were assigned a weight varying from 0 to 1, constituting a total of 8 Hamiltonian replicas. The number of replica temperatures was 24 for 2KZN and 12 for 2KW5 and 2PQX. As for temperature replica exchange, the statistical weights of the conformations are obtained by postprocessing the resulting ensemble with WHAM. The final statistical weights of the conformations are calculated for the restraint-penalty weight of 1. Details can be found in ref (53). HREMD is more efficient (but also more resource-consuming) than MREMD and, therefore, running reference MREMD simulations without time averaging was necessary to assess the effect of time averaging on the results.

**Figure 14:**
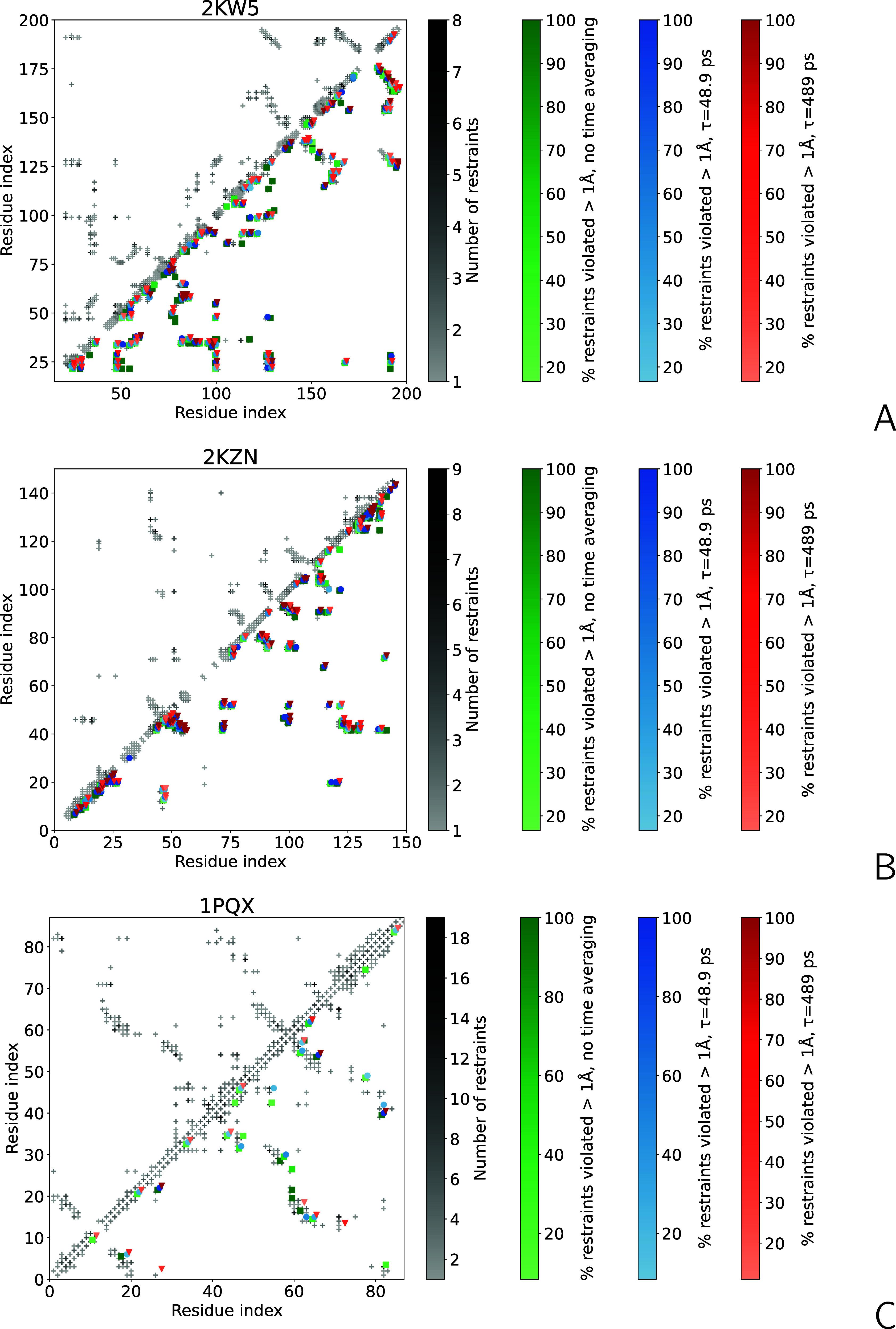
Diagrams of NMR-determined interproton-distance restraints and ensemble-averaged distance-restraint violations of the conformational ensembles obtained from MREMD simulations for (A) 2KW5, (B) 2KZN, and (C) 1PQX. The interproton-distance restraints pertaining to pairs of residues are shown as crosses in the upper-diagonal part, with the degree of gray proportional to the number of interproton-distance restraints pertaining to a pair. Restraint violations are shown in the lower-diagonal part as green circles, for the simulations without time averaging, blue squares, for the simulations with time averaging, τ = 48.9 ps, *n*_ave_ = 500, and red triangles for the simulations with time averaging, τ = 489 ps, *n*_ave_ = 1000, the color saturation proportional to the percentage of violated restraints for a given pair. Only the violations no less than 1 Å are shown. The crosses in the lower-diagonal part indicate the pairs of residues where no violations were observed.

The ρ_*u*_^+^s from the upper distance boundaries, and the percentages of satisfied distance restraints are shown in Figure 13A,B, respectively. The values obtained by averaging over the representative conformations of 20 families obtained using the procedure described in section “Calculation Procedure” and those obtained by averaging over the structures of the respective PDB ensembles are also shown in Figure 13. The numerical values of the deviations from all restraints are collected in the respective machine-readable files of the Suppinfo.zip archive. The ρ_*u*_^+^s and the numbers of violated restraints are collected in Table S1 of the Supporting Information, respectively. The diagrams of the interproton-distance restraints between pairs of residues and violations of the upper boundaries corresponding to the three calculation modes for the three proteins are shown in Figure 14A–C.

It can be seen from Figures 13 and 14 that the ρ_*u*_^+^s from the upper distance boundaries and the numbers of distance-boundary violations are smaller for the simulations with time averaging compared to those obtained in reference MREMD simulations. Only for 1PQX the ensemble obtained with τ = 48.9 ps is the ρ_*u*_^+^ higher than that from the reference simulation. However, the structure of 1PQX is well-defined by the restraints and, consequently, the ρ_*u*_^+^ and the numbers of violated restraints are small. The percentages of satisfied restraints (Figure 13B) show less regularity but they encompass only the satisfied restraints that are strictly below the upper boundaries, an entry even with an incremental violation being rejected. It can also be noted that increasing τ results in a greater number of ensemble-satisfied restraints. Even when the ensemble is reduced to 20 representative conformations the ρ_*u*_^+^ is lower and the percentage of satisfied distance restraints is greater than those from the PDB ensembles for 2KW5 and 2KZN (for the latter protein the differences are smaller). These results, as well as those obtained for 2LWA, suggest that, for multistate proteins and proteins with a significant amount of disordered regions, the advantage of extensive conformational search of the coarse-grained approach and restraint averaging overcomes the disadvantage of lower resolution inherent in a coarse-grained model.

**Figure 15:**
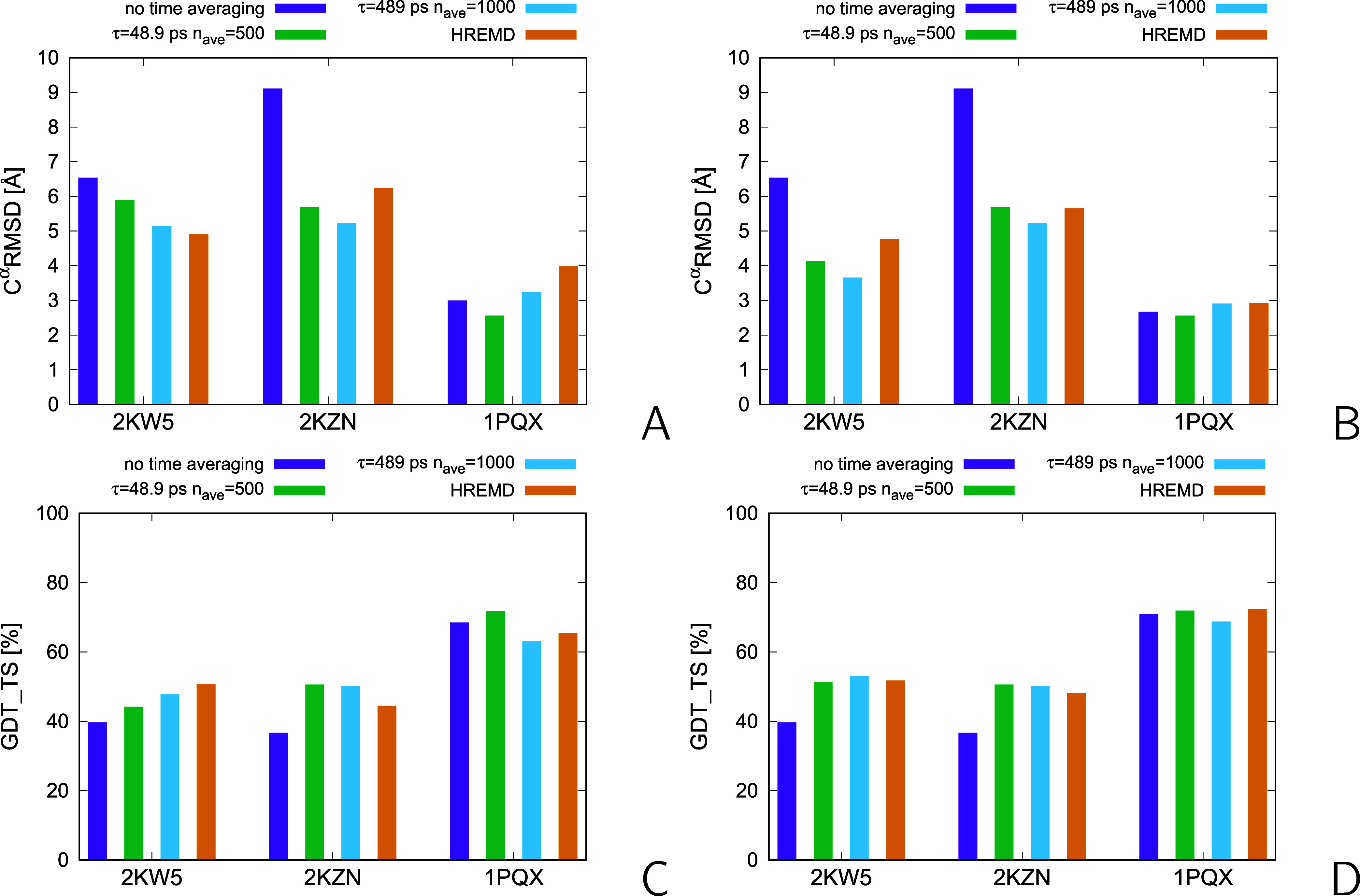
(A) Bar plot of C^α^-RMSDs from the reference X-ray structures for the top models of 1PQX, 2KW5, and 2KZN obtained in MREMD NMR-data-assisted simulations without time averaging and with time averaging, with τ = 48.9 ps, *n*_ave_ = 500, and τ = 489 ps, *n*_ave_ = 1000, respectively, and those obtained in HREMD simulations of our previous work.^53^ (B) Bar plot of the C^α^-RMSDs for the best models. (C) Bar plot of the GDT_TS of the first models (with X-ray structures as reference structures). (D) Bar plot of the GDT_TS for the best models. The plots were made with gnuplot.^79^

The RMSDs of the top and best models from the X-ray structures and the GDT_TS values are shown as bar plots in Figure 15A–D and the numerical values are collected in Table S2 of the Supporting Information, respectively. The respective structures in the PDB format are included in the Suppinfo.zip archive of the Supporting Information. For this part of the analysis, the ensembles were dissected into 5 families, as in our previous work.^53^ It can be seen that the RMSDs and GDT_TS are quite comparable for 1PQX, which again proves that the restraints define the structure of this protein well. Consequently, the resulting structure is less sensitive to the search method. On the other hand, unrestrained UNRES simulations result in a poor model of the structure of this protein, with C^α^-RMSD = 10.5 Å and GDT_TS = 28.6 (ref (53)), which demonstrates that the restraints are necessary to obtain a good model. For the two other proteins (2KW5 and 2KZN), which have disordered regions, restrained MREMD simulations without time averaging result in structures of the lowest quality, while MREMD simulations with time averaging result is structures of comparable or lower RMSD and comparable or higher GDT_TS than the more resource-consuming HREMD. This observation suggests that time averaging helps in searching the conformational space.

## Conclusions

In this work, we have implemented the time-averaged restraints in NMR-data-assisted MD with the UNRES coarse grained model of polypeptide chains. Since the presence of time-dependent terms in the potential-energy function inevitably results in energy nonconservation, we developed a stable variant of the time-averaged MD algorithm, which involves updating the time averages every *n*_ave_ steps (*n*_ave_ usually ranging from 100 or 1000) with simple averages over this time window, and using the average from the previous time windows and the current value of the conformation-dependent quantity under consideration in the other time steps (eqs 8–10). With this modification, the extended potential energy does not depend on trajectory history in each of the *n*_ave_-step time windows, which provides total-energy conservation in each of these time periods and thus prevents the average kinetic temperature from deviating remarkably from the bath temperature in canonical runs, as opposed to updating the time averages every MD step (Table 2). We also found that scaling up the average restraint forces with the ratio of the memory-window-size (τ) to the MD time step (Δ*t*) is necessary for the time-averaged restraints to affect the simulated structures (Figure 6). These two features are new with respect to the previously developed variants of the time-averaged-restraint methodology.^11−19^ Compared to the methods based on replica-averaged restraints,^21−24^ the time-averaged-restraint approach seems to provide more extensive averages because the memory-window length τ exceeds the MD time step many times (in our calculations from 1000 to 100,000 times), while the number of replicas is limited usually to several tens. However, because the NMR experiments result in both time- and ensemble-averaged observables,^9,10^ the best approach should include both time- and replica-averaging. Such an approach is now being developed in our laboratory.

With the synthetic data of the small 2KW5(129–153) system, we demonstrated that time averaging results in broadening the distribution of conformations compared to distance-restrained simulations when the restraints are derived from a single reference structure (Figure 7). When the restraints are derived from two sufficiently distinct structures, only time averaging results in the presence of structures close to the first and those close to the second reference structure in simulations (Figures 8 and 9). Moreover, the ergodicity of canonical simulations is poor without time averaging when the input restraints originate from the averages over two or more conformations.

For 2LWA, which is a multistate protein, we obtained a more diverse conformational ensemble than that deposited in the PDB.^61^ Instead of 3 distinct families of conformations present in the 2LWA PDB entry, one (structure A) characterized by a tightly packed helical hairpin, the second one (structure B) by an open helical hairpin, and the third one (structure C) by a kinked helix (Figure 3), our ensemble consists of a continuum of structures from tightly packed helical hairpins to kinked helices, with the gradually increasing angle between the two helices. Nevertheless the representative conformations of these boundary structural types constituting the PDB ensemble are present in our ensemble (Figure 10). Our ensemble results in a significantly better agreement of the calculated and experimental interproton distances than the PDB ensemble (Figure 13 and Table S1 of the Supporting Information). It should be noted that, in our coarse-grained simulations, the proton positions were estimated with ESCASA and only the final ensembles were converted to the all-atom representation from which the final interproton distances were calculated. Therefore, the agreement with the experimental interproton distances could presumably be improved if all-atom refinement subject to NMR-distance-restraints in the time-average mode was carried out.

Owing to time- and size-scale extension of coarse-grained simulations with respect to all-atom simulations, we could try modeling, with time-averaged restraints, the structures of larger proteins. We selected three proteins, one of which has a well-defined structure (1PQX), while the two other ones (2KW5 and 2KZN) have disordered regions. For 1PQX, the NMR ensemble obtained with routine data processing conforms with the experimental data better than the ensembles from our calculations. However, for the two other proteins, our calculations give better agreement with the experimental NMR restraints. Thus, when the restraints define the structure well, all-atom protein-structure modeling is advantageous over coarse-grained modeling, because the interproton distances are only estimated in the coarse-grained approach. This conclusion has already been drawn in our earlier study.^53^ However, the coarse-grained approach with time averaging seems more robust for proteins with disordered regions (Figure 13). As the results of the calculations for 2KW5 and 2KZN also suggest, introducing time averaging improves the ergodicity of simulations, because the structures obtained from time-average simulations for these two proteins had similar or higher GDT_TS and a lower or similar RMSD with respect to the reference X-ray structures (Figure 15) than those obtained in our earlier work using the more resource-intensive HREMD method.^53^ Therefore, coarse-grained data-assisted modeling with time-averaged restraints can be a method of choice for larger proteins with disordered regions, for which all-atom modeling in the time-average mode could be infeasible due to insufficient search of the conformational space.

Although the ensembles found by our NMR-data-assisted coarse-grained protein-structure-modeling approach are, except for 1PQX, in a better agreement with the experimental data than the ensembles deposited in the PDB, they contain much more conformations. Our attempts at cutting down the number of conformations to a typical NMR-determined protein-ensemble size from the PDB, by dissecting the ensemble into 20 families and selecting the best-fitting representative of each cluster resulted in ensembles still better fitting the NMR data than the corresponding PDB ensembles, except for 1PQX. Nevertheless, ensemble reweighting or limited time-average NMR-data-assisted modeling at the all-atom level are likely to result in a still better fitting. The work on this problem, as well the work on introducing explicit ϕ and ψ backbone-dihedral-angle restraints via estimating these angles from the coarse-grained geometry, is now being carried out in our laboratory.

## Data Availability

The UNRES software with time-average-modeling capacity is available at https://unres.pl/downloads under the GPL v3 license. The interproton-distance and angular restraints, the PDB files of the simulated structures, and the violations of the upper distance boundaries are in the machine-readable format in the Suppdata.zip archive, which is a part of the Supporting Information. The values of the ρ_*u*_^+^ and the numbers of upper-boundary violations are summarized in Table S1 and the C^α^-RMSD and GDT_TS values from the reference X-ray structures for the models of 1PQX, 2KW5, and 2KZN are in Table S2 of the Supporting Information.

## Supporting Information Available

Section “Glossary of the machine-readable files”. Short description of the content of the Suppdata.zip containing machine-readable files. Table S1. ρ_*u*_^+^s from the upper distance boundaries, the total numbers of violated restraints, and the numbers of restraints violated by more than 2 Å for the 2LWA, 1PQX, 2KW5, and 2KZN proteins. Table S2. C^α^-RMSD and GDT_TS values from the respective X-ray structures of the top and best models of 2KW5, 2KZN, and 1PQX obtained in NMR-data-assisted UNRES/MREMD simulations without time averaging and with time averaging (PDF)
Machine-readable files with distance and angular restraints, models of the proteins under study obtained from simulations, and NMR-restraint violations (ZIP)
