# Supplementary material for: Implementation of Time-Averaged Restraints with UNRES Coarse-Grained Model of Polypeptide Chains
Source: J Chem Theory Comput. 2025 Jan 24;21(3):1476–93. doi: 10.1021/acs.jctc.4c01504 (PMC11823420; doi:10.1021/acs.jctc.4c01504)
Supplement: Supplementary file 1 — ct4c01504_si_001.pdf [file ct4c01504_si_001.pdf]

Supporting Information for Implementation of Time-Averaged Restraints with UNRES Coarse-Grained Model of Polypeptide Chains

Nguyen Truong Co,[†] Cezary Czaplewski,[†] Emilia A. Lubecka,[‡] and Adam Liwo[*,†]

†*Faculty of Chemistry, University of Gdańsk, Fahrenheit Union of Universities, ul. Wita Stwosza 63, 80-308 Gdańsk, Poland*

‡*Faculty of Electronics, Telecommunications and Informatics, Gdańsk University of Technology, Fahrenheit Union of Universities in Gdańsk, ul. G. Narutowicza 11/12, 80-233 Gdańsk, Poland*

E-mail: adam.liwo@ug.edu.pl

Phone: +48 58 5235124. Fax: +48 58 5235012

Glossary of the machine-readable files

The machine-readable files are in the `SuppData.zip` archive, which is part of the Supporting Information. After unpacking, the archive will produce the **'SuppData'** directory. The **'SuppData'** directory contains the `README` file, the content of which is largely a plain-text version of this section and subdirectories of the following structure:

Directory 'Restraints' with subdirectories 'NMR' and 'Synthetic'

This directory contains the files with distance- and angle-restraints used in simulations. Files in the **'NMR'** subdirectory: interproton-distance and angular restraints for the 2LWA, 2KW5, 2KZN, and 1PQX proteins from NMR data extracted from the respective PDB entries. The interproton-distance data have been pruned to remove the distances pertaining to the same residue, which are not useful in coarse-grained simulations. The files PROT.nmr.dist.txt contain interproton distances, the files PROT.phipsi.txt contain original phi and psi angle restraints and the files PROT.gamma.theta.txt contain the restraints on the UNRES backbone-virtual-bond-dihedral angles gamma (the first part of a file) and on the backbone-virtual-bond angles theta (the second part of a file). PROT is the protein PDB ID. The file list is below.

Restraints		
Distance	ϕ and ψ	γ and θ
2LWA.nmr.dist.txt	2LWA.phipsi.txt	2LWA.gamma.theta.txt
2KW5.nmr.dist.txt	2KW5.phipsi.txt	2KW5.gamma.theta.txt
2KZN.nmr.dist.txt	2KZN.phipsi.txt	2KZN.gamma.theta.txt
1PQX.nmr.dist.txt	1PQX.phipsi.txt	1PQX.gamma.theta.txt

Files in the **'Synthetic'** subdirectory: coordinates of conformations #1 and #6 from which the synthetic restraints were derived and synthetic interproton-distance restraints for the 2KW5(129-153) system. The file list and meaning are below.

2KW5_129_153_1.pdb :	conformation #1 (PDB format)
2KW5_129_153_6.pdb :	conformation #6 (PDB format)

2kw5_129_153_synth_1.dist.txt :	restraints calculated from conformation #1
2kw5_129_153_synth_6.dist.txt :	restraints calculated from conformation #6
2kw5_129_153_synth_1+6.dist.txt :	average restraints from conformations #1 and #6

Directory 'Structures' with subdirectories 'Ensembles_20' and 'Models'

This directory contains the files (PDB format) with the simulated structures of 2LWA, 2KW5, 2KZN, and 1PQX.

Files in the **'Ensembles_20'** subdirectory contain the ensembles of 20 structures of the proteins listed above obtained in MREMD calculations run in different modes and clustered at $T = 280$ K by Ward's minimum-variance method into 20 families, the structures of the respective clusters best fitting NMR data selected and converted to all-atom representation by using cg2all. The filenames are PROT_20_mode.pdb where PROT is the PDB protein ID and mode is noave (no time averaging), tau_48.9ps (time averaging with $\tau = 48.9$ ps) and tau_489ps (time averaging with $\tau = 489$ ps). The REMARK record of each structure contains the free energy of the cluster it represents; these free energies have been used in the computation of the interproton distances averaged over the respective ensembles of 20 conformations.

No averaging	$\tau = 48.9$ fs	$\tau = 489$ fs
2LWA_20_noave.pdb	2LWA_20_tau_48.9ps.pdb	2LWA_20_tau_489ps.pdb
2KW5_20_noave.pdb	2KW5_20_tau_48.9ps.pdb	2KW5_20_tau_489ps.pdb
2KZN_20_noave.pdb	2KZN_20_tau_48.9ps.pdb	2KZN_20_tau_489ps.pdb
1PQX_20_noave.pdb	1PQX_20_tau_48.9ps.pdb	1PQX_20_tau_489ps.pdb

Files in the **'Models'** subdirectory contain the coarse-grained models of 2KW5, 2KZN, and 1PQX obtained after dissecting the ensembles into 5 families and selecting the representatives

best fitting the NMR data. The clustering was carried out at $T = 260$ K and $T = 280$ K. The UNRES energies and the RMSDs from the respective X-ray reference structures are in the REMARK record of each structure. The last column of each ATOM record is the uncertainty of the position of the respective C^α/SC center, estimated from the weighted standard deviation over the whole family. A filename starts with the protein ID, and contains temperature and calculation mode (without averaging, with averaging $\tau = 48.9$ ps and $\tau = 489$ ps, respectively, as for the 'Ensembles_20' files).

Clustering temperature T = 260 K		
No averaging	τ = 48.9 fs	τ = 489 fs
2KW5_T260K_noave-cg.pdb	2KW5_T260K_tau_48.9ps-cg.pdb	2KW5_T260K_tau_489ps-cg.pdb
2KZN_T260K_noave-cg.pdb	2KZN_T260K_tau_48.9ps-cg.pdb	2KZN_T260K_tau_489ps-cg.pdb
1PQX_T260K_noave-cg.pdb	1PQX_T260K_tau_48.9ps-cg.pdb	1PQX_T260K_tau_489ps-cg.pdb
Clustering temperature T = 280 K		
No averaging	τ = 48.9 fs	τ = 489 fs
2KW5_T280K_noave-cg.pdb	2KW5_T280K_tau_48.9ps-cg.pdb	2KW5_T280K_tau_489ps-cg.pdb
2KZN_T280K_noave-cg.pdb	2KZN_T280K_tau_48.9ps-cg.pdb	2KZN_T280K_tau_489ps-cg.pdb
1PQX_T280K_noave-cg.pdb	1PQX_T280K_tau_48.9ps-cg.pdb	1PQX_T280K_tau_489ps-cg.pdb

Directory ‘Violations’

This directory contains the files with the experimental upper distance boundaries and right deviations from the upper boundaries (0 if the actual distance does not exceed the upper boundary) for the simulated ensembles of 2LWA, 2KW5, 2KZN, and 1PQX and the deviations calculated for the respective PDB ensembles. The filenames are PROT_distviol.txt, where PROT is protein PDB ID and the headers explain the meaning of the respective columns. The file list is below.

2LWA_distviol.txt

2KW5_distviol.txt

2KZN_distviol.txt

1PQX_distviol.txt

Table S1: Right RMSDs from the upper distance boundaries (ρ_u^+s; eq 17 of the main text), the total numbers of violated restraints (n_{viol}), and the numbers of restraints violated by more than 2 Å (N_{viol}) for the 2LWA, 1PQX, 2KW5, and 2KZN proteins.

Protein	Calc. mode	ESCASA[a]			all-atom whole ensemble[b]			all-atom 20 families[c]			PDB[d]		
		ρ_u^+	n_{viol}	N_{viol}	ρ_u^+	n_{viol}	N_{viol}	ρ_u^+	n_{viol}	N_{viol}	ρ_u^+	n_{viol}	N_{viol}
2LWA	no ave.	0.49	50	3	0.41	30	2	0.65	41	4			
	$\tau = 48.9$ ps	0.30	35	0	0.11	20	0	0.30	30	0	0.44	41	1
	$\tau = 489$ ps	0.21	28	0	0.10	17	0	0.28	30	1			
2KW5	no ave.	0.51	329	7	0.40	227	5	0.58	283	17			
	$\tau = 48.9$ ps	0.46	225	8	0.26	189	1	0.50	295	6	0.66	423	21
	$\tau = 489$ ps	0.46	182	8	0.25	166	0	0.44	286	4			
2KZN	no ave.	0.62	196	12	0.51	149	7	0.68	228	13			
	$\tau = 48.9$ ps	0.53	172	9	0.42	177	4	0.76	244	19	0.61	227	9
	$\tau = 489$ ps	0.45	120	7	0.31	153	2	0.60	204	11			
1PQX	no ave.	0.14	55	0	0.11	33	0	0.20	51	3			
	$\tau = 48.9$ ps	0.22	54	3	0.18	22	3	0.36	63	10	0.02	6	0
	$\tau = 489$ ps	0.10	34	0	0.07	20	0	0.26	53	4			

[a]Values estimated by ESCASA.[1]

[b]Values calculated after conversion to all-atom representation with `cg2all`.[2,3]

[c]Values calculated from the representatives of 20 families after minimum-variance dissection of the respective ensemble.

[d]Values calculated from the respective PDB ensembles.

Table S2: C^α-RMSD and GDT_TS values from the respective X-ray structure of the first-rank-clusters (first) and lowest-RMSD (best) models of 2KW5, 2KZN, and 1PQX obtained in NMR-data-assisted UNRES/MREMD simulations without and with time averaging (with τ = 48.9 and 489 fs, respectively, and those obtained without time averaging by using Hamiltonian Replica Exchange MD (HREMD) in ref 4.

Protein	Calc. mode	First		Best	
		RMSD	GDT_TS	RMSD	GDT_TS
2KW5	no ave.	6.53	39.6	6.53	39.6
	τ = 48.9 fs	5.88	44.1	4.13	51.3
	τ = 489 fs	5.14	47.7	3.65	52.9
	HREMD[a]	4.90	50.6	4.76	51.7
2KZN	no ave.	9.10	36.6	9.10	36.6
	τ = 48.9 fs	5.68	50.5	5.68	50.5
	τ = 489 fs	5.22	50.1	5.22	50.1
	HREMD[a]	6.23	44.4	5.65	48.1
1PQX	no ave.	2.99	68.4	2.66	70.8
	τ = 48.9 fs	2.55	71.7	2.55	71.8
	τ = 489 fs	3.24	63.0	2.90	68.7
	HREMD[a]	3.98	65.4	2.92	72.3

[a]Values from reference 4.

References

(1) Lubecka, E.; Liwo, A. ESCASA: Analytical Estimation of Atomic Coordinates from Coarse-Grained Geometry for Nuclear-Magnetic-Resonance-Assisted Protein Structure Modeling. I. Backbone and H^β Protons. *J. Comput. Chem.* **2021**, *42*, 1579–1589.

(2) Heo, L.; Feig, M. One Bead per Residue Can Describe All-Atom Protein Structures. `https://github.com/huhlim/cg2all`, 2023; cg2all version v1.3.1.

(3) Heo, L.; Feig, M. One Bead Per Residue Can Describe All-Atom Protein Structures. *Structure* **2024**, *32*, P97–111.E6.

(4) Lubecka, E.; Liwo, A. A Coarse-Grained Approach to NMR-Data-Assisted Modeling of Protein Structures. *J. Comput. Chem.* **2022**, *43*, 2047–2059.
